# The association of sodium‐glucose cotransporter 2 inhibitors with cancer: An overview of quantitative systematic reviews

**DOI:** 10.1002/edm2.145

**Published:** 2020-05-20

**Authors:** Ryan Pelletier, Kelvin Ng, Wajd Alkabbani, Youssef Labib, Nicolas Mourad, John‐Michael Gamble

**Affiliations:** ^1^ School of Pharmacy Faculty of Science University of Waterloo Kitchener ON Canada

**Keywords:** adverse events, cancer, overview of reviews, SGLT2 inhibitors, type 2 diabetes, umbrella reviews

## Abstract

**Aims:**

To summarize reported cancer events associated with SGLT‐2 inhibitors used in patients with type 2 diabetes mellitus, as well as assess the quality of included reviews.

**Materials and methods:**

In May 2019, we searched PubMed, Embase and the Cochrane Library for quantitative systematic reviews assessing the safety of SGLT‐2 inhibitors. Data were abstracted using a standardized form, and methodological quality was assessed using the AMSTAR 2 tool. Main outcome measures included total cancer events and specific cancers such as breast cancer, bladder cancer, gastrointestinal cancer, prostate cancer, respiratory cancer, renal cancer and skin cancer. Pooled treatment effects from included reviews were summarized for SGLT‐2 inhibitors as a class and for individual SGLT‐2 inhibitors commonly used worldwide (canagliflozin, dapagliflozin and empagliflozin).

**Results:**

We screened 1248 unique citations, of which eight quantitative systematic reviews meta‐analysed results from studies reporting the association between an SGLT‐2 inhibitor and any cancer. Only one review was rated as high quality according to AMSTAR 2 assessment. In total, data from 170 cancer‐related point estimates (PE) were reported. As a class, SGLT‐2 inhibitors were not associated with an increased risk of any cancer event versus placebo and active comparators. Most point estimates (7/143) were nonsignificant for individual cancers except for two associations. Empagliflozin was associated with an increased risk of bladder cancer versus placebo and active comparators in two reviews, while canagliflozin appeared protective for gastrointestinal cancer versus placebo and active comparators in one review.

**Conclusions:**

It appears that SGLT‐2 inhibitors are not associated with an increased risk of total cancer or specific cancers in patients with type 2 diabetes. However, higher quality evidence is needed to derive confident conclusions.

## Graphical abstract

The association of sodium glucose cotransporter 2 (SGLT2) inhibitors with cancer has been reported in several quantitative systematic reviews. Most quantitative systematic reviews addressing cancer risk in patients with type 2 diabetes mellitus are of low quality (according to AMSTAR2 assessment). From the available evidence, it appears that SGLT2 inhibitors are not associated with an increased or decreased risk of total cancer events, or specific cancers, in patients with type 2 diabetes.

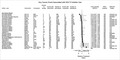

## 1 INTRODUCTION

Sodium‐glucose cotransporter 2 (SGLT‐2) inhibitors are a novel class of antihyperglycaemic agents used in the treatment of type 2 diabetes mellitus. These agents inhibit the SGLT‐2 protein expressed in the proximal tubule within the kidney, which is responsible for the renal reabsorption of glucose.^1^ Inhibition of these transporters facilitates blood glucose reduction via urinary excretion of glucose.^1^ There are a wide variety of benefits associated with SGLT‐2 inhibitor use in type 2 diabetes, including significant reduction in haemoglobin A1C, reduction in major cardiovascular adverse events (MACE) and significant reduction in the risk of end‐stage kidney disease compared to placebo.^2^, ^3^, ^4^ Due to these demonstrated benefits, the utilization of SGLT‐2 inhibitors for the treatment of type 2 diabetes mellitus has rapidly increased since market approval.^5^ However, these agents have undergone unprecedented postmarketing investigations given the FDA requirements to demonstrate cardiovascular safety of new antihyperglycaemic agents. Despite elusive mechanisms, cancer risk associated with SGLT‐2 inhibitors has been reported in several quantitative systematic reviews.^6^, ^7^, ^8^, ^9^, ^10^, ^11^, ^12^, ^13^

There are signals in the literature that SGLT‐2 inhibitors may affect cancer risk. It has been postulated that SGLT‐2 inhibitors may activate medullary thyroid tumour growth in both rats and male mice; however, the relevance of this information in humans is not known.^14^, ^15^ Furthermore, in 2011, the US Food and Drug Administration (FDA) observed discrepancies in the risk of bladder and breast cancers with dapagliflozin versus comparators.^16^ Regulatory concerns were also raised due to an imbalance of lung cancer and melanoma observed with empagliflozin use.^17^ In contrast, canagliflozin has been associated with a decreased risk of stomach cancer.^10^ SGLT‐1 has been implicated in cancer cell survival via glucose uptake; therefore, canagliflozin's inhibition of both SGLT‐1 and SGLT‐2 receptors has been proposed for this agent's purported protective effect.^18^

Interestingly, there have been several systematic reviews and meta‐analyses reporting on cancer risk associated with SGLT‐2 inhibitor use. A combination of low cancer event rates, poor diagnostic consistency and short follow‐up times of studies included in quantitative reviews assessing cancer risk in SGLT‐2 inhibitor users thus far have made it difficult for clinicians to draw confident conclusions on potentially relevant implications of this data in practice. Given these limitations as well as variance in the methodological rigour of published quantitative systematic reviews, there is a need to critically review, evaluate and summarize these studies. Therefore, we conducted an overview of reviews, adapted from Cochrane Overviews, which serves to effectively accomplish this task.^19^ An overview of reviews provides clinicians, policymakers and clinical guideline developers with a summary of the available evidence for a topic of interest. We aimed to summarize evidence from and assess the quality of published quantitative systematic reviews evaluating the cancer risk associated with SGLT‐2 inhibitor use in the treatment of type 2 diabetes.

## 2 METHODS

The protocol for this overview of reviews is registered with the PROSPERO international prospective register of systematic reviews (PROSPERO 2019:CRD42019135863).^20^ This overview is part of a series of overviews of reviews exploring various adverse events associated with SGLT‐2 inhibitor use in patients with type 2 diabetes mellitus.

### 2.1 Eligibility criteria

Systematic reviews of randomized controlled studies, cohort or case‐control studies with a meta‐analysis (ie quantitative systematic reviews) that evaluated SGLT‐2 inhibitor safety and collected data on adverse events (beyond hypoglycaemia) were included. Quantitative systematic reviews that did not use a systematic search strategy were excluded. Our outcomes of interest were any point estimates reporting on the association between SGLT‐2 inhibitors and any type of cancer in quantitative systematic reviews. We did not restrict the inclusion of quantitative systematic reviews based on the timing of the outcome following drug exposure. We restricted the language of included reviews to English.

### 2.2 Sources and searching

Potentially relevant quantitative systematic reviews were identified through a comprehensive search of bibliographic electronic databases and other sources. First, we searched the following databases: PubMed, Embase and the Cochrane Library from inception to 15 May 2019. A systematic review filter was used within the search strategy where applicable. Second, we searched the table of contents from the following diabetes journals from 1 January 2011 to 15 May 2019: *Diabetes Care, Diabetologia, Diabetic Medicine, Diabetes Research and Clinical Practice, Diabetes, Obesity and Metabolism, Diabetes* and *The Lancet Diabetes and Endocrinology*. Third, we hand searched the references of included systematic reviews. The search strategy is available in Appendix S1.

### 2.3 Study selection

**FIGURE 1:**
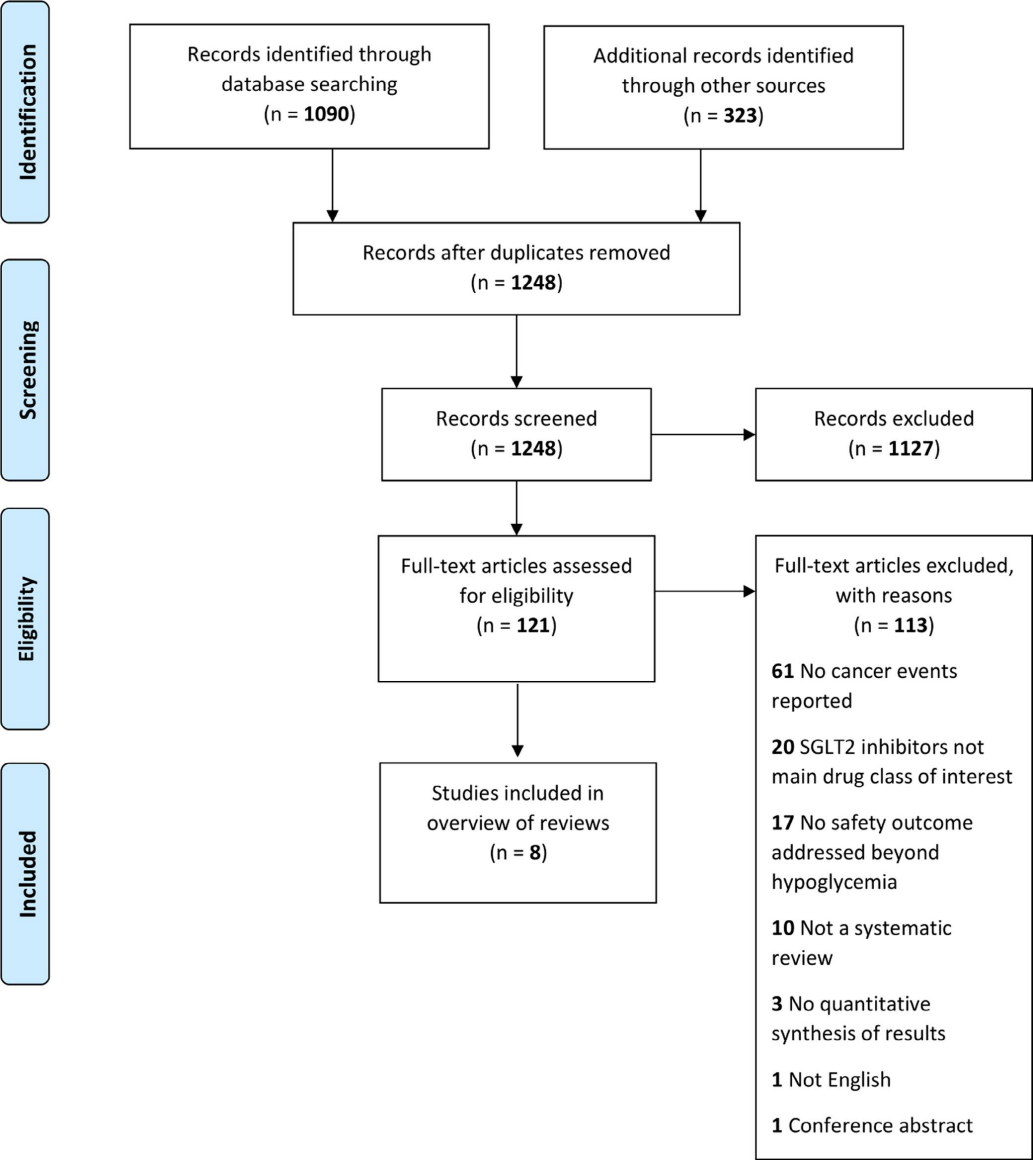
Flow diagram of study selection

Two independent reviewers (RP, KN, WA, YL, NM, JMG) screened the titles and abstracts of all citations identified by the search strategy. Using a standardized study eligibility form, two independent reviewers (RP, KN, WA, YL, NM, JMG) further reviewed the full texts of citations that were potentially relevant. Disagreements were resolved by consensus or by a third reviewer (JMG). Study selection is summarized in Figure 1.

### 2.4 Data extraction

One reviewer (RP, KN, WA, YL, NM, JMG) extracted relevant review‐level data from the eligible quantitative systematic reviews and recorded it on a standardized Google Form developed for the present overview. Information was extracted from each included quantitative systematic review on bibliographic details, research question(s)/objective(s), search strategies, number of included studies, interventions and comparisons evaluated, outcomes reported and methods of analysis used. Two reviewers (RP, KN, WA, YL, NM) extracted all pooled and single study estimates from each included review, and verification of all estimates was completed through consensus. We extracted pooled estimates calculated from traditional pairwise meta‐analytical techniques, as well as indirect and mixed treatment point estimates from network meta‐analytical techniques.

### 2.5 Quality assessment

Two independent reviewers assessed the quality of included systematic reviews using the ‘A MeaSurement Tool to Assess systematic Reviews 2’ (AMSTAR 2) checklist.^21^ AMSTAR 2 is a validated tool consisting of 16 domains that assess the methodological quality of systematic reviews containing both randomized and nonrandomized studies of interventions. All discordant AMSTAR 2 quality ratings between reviewers were resolved by consensus. Consistent with AMSTAR 2 published literature, systematic reviews having more than one critical flaw were rated as critically low quality, one critical flaw as low quality, more than one noncritical weakness as moderate quality and no or one noncritical weakness as high quality. Domains 2, 7, 4, 9, 11, 13 and 15 are considered critical in AMSTAR 2.^21^

### 2.6 Analysis

We conducted a descriptive analysis of our results by summarizing the bibliographic characteristics of included quantitative systematic reviews, as well as by summarizing the point estimates for each adverse outcome assessed. We tabulated the number of systematic reviews and number of pooled estimates of treatment effect for all placebo and active treatment comparisons for SGLT‐2 inhibitors as a class, as well as individual SGLT‐2 inhibitors used commonly worldwide (ie canagliflozin, dapagliflozin and empagliflozin). We used forest plots to report pooled point estimates and 95% confidence intervals (CIs) from included systematic reviews for all cancer outcomes. Furthermore, we plotted pooled estimates from reviews according to individual SGLT‐2 inhibitor agents, as well as concomitant treatment with background antihyperglycaemic agents.

## 3 RESULTS

We identified 1248 unique citations, of which eight quantitative systematic reviews met our inclusion criteria (Table 1). Four reviews (50%) reported no funding source, while one review (12.5%) received funding from government, one review (12.5%) received internal funding and one review (12.5%) received foundational funding. A funding source was not disclosed in one review (12.5%). The median (interquartile range [IQR]) number of databases searched was four (1). The median (IQR) number of studies included was 32 (21.5). There were 170 cancer‐related point estimates reported by the eight included reviews, whereby the most frequently reported estimates (16%) were for any cancer event. There were also 143 point estimates reported for 11 specific types of cancers.

**TABLE 1:** Characteristics of included systematic reviews and meta‐analyses

First author name (year)	Protocol	Country	Funding source	Intervention(s)	Comparator(s)	Cancer outcome(s)	Data synthesis model used	Number of studies meta‐analysed
Wilbert Aronow^9^ (2017)	Yes, not published (a priori)	USA	Foundational	SGLT‐2 inhibitor: empagliflozin	Placebo; active (non‐SGLT‐2 inhibitors)	Bladder cancer	Random effects model	30
Ilaria Dicembrini^13^ (2019)	Yes	Italy	None	SGLT2 inhibitors (canagliflozin 100 mg, 300 mg; dapagliflozin 5 mg, 10 mg; empagliflozin 10 mg, 25 mg; ertugliflozin 5 mg, 15 mg; ipragliflozin 25 mg, 50 mg; luseogliflozin 2.5 mg, 5 mg; tofogliflozin 20 mg)	Placebo; active drugs (non‐SGLT‐2 inhibitors)	Any cancer event; breast cancer; bladder cancer; gastrointestinal cancer; prostate cancer; respiratory cancer; renal cancer; skin cancer; pancreatic cancer; female genital tract cancer; hepatic cancer	Random effects model	27
Matteo Monami^6^ (2014)	No	Italy	None	SGLT‐2 inhibitors^a^; (canagliflozin; dapagliflozin; empagliflozin; ipragliflozin)	Placebo; active drugs (oral antihyperglycaemic agents and/or insulin)	Any cancer event (malignancies)	Random effects model	25
Karin Radholm^11^ (2018)	No	Sweden	Not disclosed	SGLT‐2 inhibitors^a^ (canagliflozin; dapagliflozin; empagliflozin; ipragliflozin; luseogliflozin; tofogliflozin; ertugliflozin)	Placebo; sitagliptin; glimepiride; metformin; sulfonylurea; exenatide; placebo/sitagliptin; placebo/HCTZ	Any cancer event (total); breast cancer; bladder cancer; renal cancer (kidney)	Fixed effect model	82 studies, 4 overviews, 6 regulatory reports
Heidi Storgaard^8^ (2016)	Yes	Denmark	None	Canagliflozin 300 mg; dapagliflozin 10 mg; empagliflozin 25 mg	Placebo; glimepiride 8 mg; sitagliptin 100 mg; metformin 1500 mg; metformin 2000 mg; glipizide 20 mg; saxagliptin 5 mg; linagliptin 5 mg; glimepiride 1‐4 mg	Cancer (other than Bladder or Breast)	Random effects model	42
Huilin Tang^10^ (2017)	No	China	Internal	SGLT‐2 inhibitors; (canagliflozin; dapagliflozin; empagliflozin)	Placebo or other glucose‐lowering treatments	Any cancer event (Overall); breast cancer; bladder cancer; gastrointestinal cancer; prostate cancer; pulmonary cancer (respiratory); renal cancer; skin cancer	Random effects model	45 studies with 46 independent RCTs
Huilin Tang^12^ (2018)	No	USA	None	SGLT‐2 inhibitors; (canagliflozin; dapagliflozin; empagliflozin; ertugliflozin; ipragliflozin)	Placebo or other antidiabetic drugs	Melanoma skin cancer; nonmelanoma skin cancer	Random effects model	21
Jason Wu^7^ (2018)	No	Australia	Government	SGLT‐2 inhibitors; (canagliflozin; dapagliflozin; empagliflozin; ipragliflozin; luseogliflozin; tofogliflozin)	Placebo or other antidiabetic drugs	Any cancer event	Fixed effect model	57 studies and 6 regulatory submissions

^a^ Studies that did not specifically list doses for their intervention.

### 3.1 Quality assessment

The complete AMSTAR 2 assessments and overall quality ratings for included systematic reviews are shown in Appendix S1: Figure S1. Only one (12.5%) included review received an AMSTAR 2 quality rating of high.^8^ Four (50%) reviews were considered critically low quality, one (12.5%) review was considered low quality and two (25%) reviews were considered moderate quality.

### 3.2 Any cancer event

**FIGURE 2:**
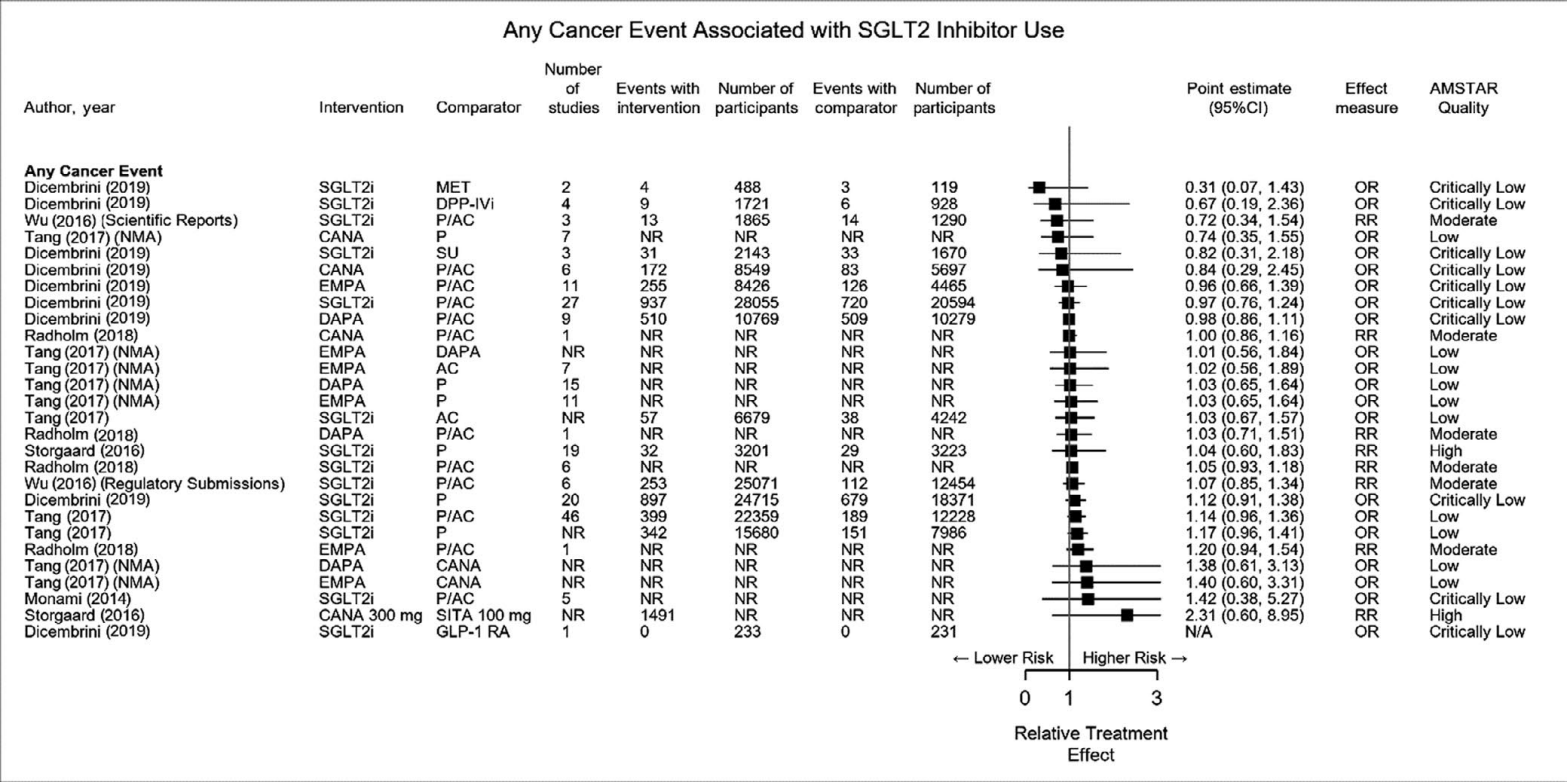
Any cancer event associated with sodium‐glucose cotransporter 2 use. NMA, network meta‐analysis; NR, not reported; SGLT2i, sodium‐glucose cotransporter 2 inhibitors (class effect)

From the eight included reviews, 27 point estimates were reported for the risk of any cancer event with SGLT‐2 inhibitors as a class vs placebo and active comparators, including estimates reported for individual SGLT‐2 inhibitor agents (Figure 2). SGLT‐2 inhibitors were not associated with an increased risk of any cancer event versus placebo or active comparators (point estimate range 0.72‐1.42; *P* > .05 for all). Likewise, canagliflozin, dapagliflozin and empagliflozin were not associated with an increased risk of any cancer event versus placebo or active comparators (point estimate range 0.74‐1.40; *P* > .05 for all).

### 3.3 Site‐specific cancers

A total of 143 point estimates were reported for 11 specific types of cancers (Appendix S2: Figures S1‐S8). There were 26 point estimates from three reviews for skin cancer, 21 point estimates from four reviews were reported for bladder cancer, 21 point estimates from four reviews were reported for breast cancer, 18 point estimates from four reviews were reported for renal cancer, 15 point estimates from two reviews were reported for gastrointestinal cancer, 15 point estimates from two reviews were reported for prostate cancer, 15 point estimates from two reviews were reported for pulmonary cancer, four point estimates from one review were reported for pancreatic cancer, four point estimates from one review were reported for hepatic cancer, three point estimates from one review were reported for female genital tract cancer, and one point estimate from one review was reported for cancers ‘other than bladder or breast’.

From all the point estimates reported for site‐specific cancers, seven were considered statistically significant. Two point estimates from one review indicated a significantly increased class association of bladder cancer with SGLT‐2 inhibitors (OR 3.87, 95%CI 1.48‐10.08 versus placebo and active comparators; OR 3.71, 95%CI 1.38‐9.96 vs placebo). Additionally, two point estimates from two different reviews reported a statistically significant increased association of bladder cancer with empagliflozin (OR 4.49, 95%CI 1.21‐16.73 versus placebo and active comparators; OR 7.37, 95%CI 1.28‐42.59 vs placebo). Two point estimates from one review indicated a significantly decreased association of gastrointestinal cancer with canagliflozin (OR 0.15, 95%CI 0.04‐0.60 vs placebo and active comparators; OR 0.31, 95%CI 0.11‐0.88 vs placebo). A significantly increased association of gastrointestinal cancer with empagliflozin vs canagliflozin (OR 4.01, 95%CI 1.34‐11.96) was reported by a network meta‐analysis.^10^

## 4 DISCUSSION

SGLT‐2 inhibitors do not appear to be associated with an overall increased risk of cancer in patients with type 2 diabetes mellitus. Point estimates reported for class effects of SGLT‐2 inhibitors on the risk of any cancer event, as well as specific cancer subtypes, showed no significant association with the use of these agents. This held true regardless of whether SGLT‐2 inhibitor interventions were compared with placebo or active comparators. However, practicing clinicians do not prescribe by class, but rather by individual SGLT‐2 inhibitor agents for antihyperglycaemic management. Considering popular SGLT‐2 inhibitor agents used globally (ie canagliflozin, dapagliflozin and empagliflozin), most cancer‐related data collected for these individual agents also indicated there were no significant associations between their use and overall risk of any cancer event. Some individual point estimates from included reviews, specifically for canagliflozin and empagliflozin, reported a statistically significant decreased risk of gastric cancer and increased risk of bladder cancers for users of these agents, respectively.^10^, ^13^

There are several potential reasons that could account for the statistically significant associations observed between empagliflozin use and bladder cancer. First, detection bias is a plausible explanation for this increased risk.^22^ SGLT‐2 inhibitors may increase the risk of genital tract infections secondary to their mechanism of action^23^, ^24^, ^25^; however, investigation into these infections (eg urinalysis) may prompt further diagnostic workup and eventual diagnosis of bladder cancers that were present before initiation of SGLT‐2 inhibitor therapy. Second, cautious interpretation is warranted as the observed association is driven by the imbalance between empagliflozin and comparator users in a very lower numbers of events. In fact, there were zero events in each comparator group for the reported significant point estimates. Third, bladder cancer pathogenesis follows an insidious course over several years for most cases, and follow‐up beyond one year was rare in both randomized and nonrandomized studies assessed in our included systematic reviews. One quantitative systematic review excluded studies that had participant follow‐up of less than one year. This review accounted for 48 cases of bladder cancer in 28 055 participants treated with SGLT‐2 inhibitors, compared to 58 cases of bladder cancer in 20 594 participants treated with placebo or active comparators.^13^ Lastly, it is possible that prolonged bladder irritation due to recurrent or chronic urinary tract infections increases the risk of bladder cancer; however, the current evidence is unreliable and does not demonstrate a causal association between empagliflozin and an increased risk of bladder cancer.

Additionally, evidence from a 2017 meta‐analysis by Tang et al suggested a statistically significant decreased risk of gastrointestinal cancer in canagliflozin users.^10^ As noted previously, these results should be interpreted with caution, as short follow‐up times of included randomized controlled trials and low event rates preclude evaluation of long‐term gastrointestinal cancer risk in participants using canagliflozin. Furthermore, this association was not evident from a meta‐analysis conducted by Dicembrini et al in 2019.^13^

Although several quantitative systematic reviews have been published assessing cancer‐related events as primary and secondary outcomes in SGLT‐2 inhibitor users, the methodological rigour of these studies appears to be inconsistent. Half of the included quantitative systematic reviews were considered to be of ‘critically low quality’ according to AMSTAR 2 assessment. Since systematic reviews are considered to be at the top of the scientific evidence pyramid, it is crucial that the methods undertaken to complete these reviews are transparent and replicable.^26^ However, this does not necessarily mean that the point estimates reported within reviews deemed ‘low quality’ by AMSTAR 2 rating are sourced from low quality evidence. The quality of evidence contained within the included reviews was generally high (ie evidence from randomized controlled trials and government regulatory reports). It is important to remember that the AMSTAR 2 tool is used to measure the methodological quality of systematic reviews, not to assess the quality of evidence contained within the review.

Our review provides clinicians with a comprehensive summary that highlights important limitations of assessing SGLT‐2 inhibitor‐associated cancer risk using quantitative systematic reviews. Despite using established methods (eg published protocol, comprehensive search strategy, screening and quality assessment performed by at least 2 independent reviewers), our overview also has some limitations. We did not meta‐analyse the point estimates gathered from included reviews as this was beyond the scope of this study. Additionally, our unit of analysis was at the review level. Furthermore, with the large volume of reviews that have been published on SGLT‐2 inhibitor safety, it is possible that additional studies and reviews assessing cancer risk in our population of interest are currently under consideration for publication. A potential resolution to prevent evidence from individual systematic reviews from becoming quickly outdated is to develop a ‘living systematic review’ that has been described by the Cochrane community.^27^ With an updated literature search that is ideally conducted once monthly, living systematic reviews are continually updated with the most current evidence as it becomes available.

## 5 CONCLUSION

As current evidence stands, canagliflozin, dapagliflozin and empagliflozin do not appear to significantly impact cancer risk in patients with type 2 diabetes; however, long‐term safety data are lacking. Given the limitations of the included quantitative systematic reviews, as well as imprecise effect estimates reported in these reviews, more long‐term data from high quality observational studies are needed to more precisely assess cancer risks associated with SGLT‐2 inhibitor use. Future studies should focus on quantifying bladder and gastrointestinal cancers.

## CONFLICT OF INTEREST

None of us have any financial arrangements or any potential conflicts of interest to disclose with regard to the products in this manuscript.

## AUTHOR CONTRIBUTION

RP, KN and JMG conceptualized the review. RP wrote the first draft of the manuscript, made suggested changes from co‐authors and formatted the paper for publication. All authors partook in the review selection and critical appraisal processes, as well as provided intellectual feedback on manuscript drafts. All authors approved the final draft of the manuscript prior to submission.

## ETHICS STATEMENT

Our study did not undergo review by a human research ethics board as it did not involve human subjects and consisted of a review of the literature using aggregated anonymous data.

## Supporting information

Appendix S1

Appendix S2

## Data Availability

Data are available within the article and its Appendix S1 and Appendix S2.
