# Supplementary material for: The association of sodium‐glucose cotransporter 2 inhibitors with cancer: An overview of quantitative systematic reviews
Source: Endocrinol Diabetes Metab. 2020 May 20;3(3):e00145. doi: 10.1002/edm2.145 (PMC7375059; doi:10.1002/edm2.145)
Supplement: Supplementary file 1 — Appendix S1 [file EDM2-3-e00145-s001.docx]

**Appendix 1:**

**Page 2.** PubMed search strategy.

**Pages 3-4.** EMBASE search strategy

**Page 5.** Cochrane Library search strategy

**Pages 6-19.** Table S1. Excluded studies and reasons for exclusion, with references.

**Page 20.** Figure S1. A MeaSurement Tool to Assess systematic Reviews 2 (AMSTAR 2) quality assessment of included quantitative systematic reviews.

**PubMed Search Strategy**

(diabetes mellitus, type 2[mesh] OR type 2 diabet* OR type II diabet*) 

AND

(Sodium-glucose transporter 2[mesh] OR SGLT-2 Inhibitor* OR Sodium-glucose co-transporter-2 inhibitor* OR sodium glucose linked transporter 2 inhibitor OR canagliflozin OR canagliflozin[mesh] OR Invokana OR Invokamet OR dapagliflozin OR Forxiga OR Xigduo OR empagliflozin OR Jardiance OR Synjardy OR ertugliflozin OR Stelagtro OR Segluromet OR atigliflozin OR bexagliflozin OR ipragliflozin OR Suglat OR luseogliflozin OR Lusefi OR remogliflozin OR Sergliflozin OR tofogliflozin OR Apleway OR Deberza)

AND

Systematic review OR meta-analysis (filters)

**EMBASE Search Strategy**

1 exp sodium glucose cotransporter 2 inhibitor/ (6732)

2 sodium-glucose cotransporter 2 inhibitor.mp. [mp=title, abstract, heading word, drug trade name, original title, device manufacturer, drug manufacturer, device trade name, keyword, floating subheading word, candidate term word] (2695)

3 SGLT2 inhibitor*.mp. [mp=title, abstract, heading word, drug trade name, original title, device manufacturer, drug manufacturer, device trade name, keyword, floating subheading word, candidate term word] (2719)

4 sodium glucose linked transporter 2 inhibitor.mp. [mp=title, abstract, heading word, drug trade name, original title, device manufacturer, drug manufacturer, device trade name, keyword, floating subheading word, candidate term word] (7)

5 canagliflozin.mp. [mp=title, abstract, heading word, drug trade name, original title, device manufacturer, drug manufacturer, device trade name, keyword, floating subheading word, candidate term word] (2074)

6 exp canagliflozin/ (1983)

7 invokana.mp. [mp=title, abstract, heading word, drug trade name, original title, device manufacturer, drug manufacturer, device trade name, keyword, floating subheading word, candidate term word] (145)

8 invokamet.mp. [mp=title, abstract, heading word, drug trade name, original title, device manufacturer, drug manufacturer, device trade name, keyword, floating subheading word, candidate term word] (23)

9 exp dapagliflozin/ (2298)

10 dapagliflozin.mp. [mp=title, abstract, heading word, drug trade name, original title, device manufacturer, drug manufacturer, device trade name, keyword, floating subheading word, candidate term word] (2437)

11 forxiga.mp. [mp=title, abstract, heading word, drug trade name, original title, device manufacturer, drug manufacturer, device trade name, keyword, floating subheading word, candidate term word] (77)

12 xigduo.mp. [mp=title, abstract, heading word, drug trade name, original title, device manufacturer, drug manufacturer, device trade name, keyword, floating subheading word, candidate term word] (26)

13 exp empagliflozin/ (2149)

14 empagliflozin.mp. [mp=title, abstract, heading word, drug trade name, original title, device manufacturer, drug manufacturer, device trade name, keyword, floating subheading word, candidate term word] (2301)

15 jardiance.mp. [mp=title, abstract, heading word, drug trade name, original title, device manufacturer, drug manufacturer, device trade name, keyword, floating subheading word, candidate term word] (96)

16 synjardy.mp. [mp=title, abstract, heading word, drug trade name, original title, device manufacturer, drug manufacturer, device trade name, keyword, floating subheading word, candidate term word] (17)

17 exp atigliflozin/ (1)

18 atigliflozin.mp. [mp=title, abstract, heading word, drug trade name, original title, device manufacturer, drug manufacturer, device trade name, keyword, floating subheading word, candidate term word] (1)

19 bexagliflozin.mp. (2)

20 exp bexaglifozin/ (2)

21 exp ipragliflozin/ (387)

22 ipragliflozin.mp. [mp=title, abstract, heading word, drug trade name, original title, device manufacturer, drug manufacturer, device trade name, keyword, floating subheading word, candidate term word] (391)

23 suglat.mp. [mp=title, abstract, heading word, drug trade name, original title, device manufacturer, drug manufacturer, device trade name, keyword, floating subheading word, candidate term word] (15)

24 exp luseogliflozin/ (228)

25 luseogliflozin.mp. [mp=title, abstract, heading word, drug trade name, original title, device manufacturer, drug manufacturer, device trade name, keyword, floating subheading word, candidate term word] (232)

26 lusefi.mp. [mp=title, abstract, heading word, drug trade name, original title, device manufacturer, drug manufacturer, device trade name, keyword, floating subheading word, candidate term word] (14)

27 remogliflozin.mp. [mp=title, abstract, heading word, drug trade name, original title, device manufacturer, drug manufacturer, device trade name, keyword, floating subheading word, candidate term word] (118)

28 exp remogliflozin etabonate/ (100)

29 exp sergliflozin etabonate/ (75)

30 sergliflozin.mp. [mp=title, abstract, heading word, drug trade name, original title, device manufacturer, drug manufacturer, device trade name, keyword, floating subheading word, candidate term word] (105)

31 exp tofogliflozin/ (225)

32 tofogliflozin.mp. [mp=title, abstract, heading word, drug trade name, original title, device manufacturer, drug manufacturer, device trade name, keyword, floating subheading word, candidate term word] (227)

33 apleway.mp. [mp=title, abstract, heading word, drug trade name, original title, device manufacturer, drug manufacturer, device trade name, keyword, floating subheading word, candidate term word] (11)

34 deberza.mp. [mp=title, abstract, heading word, drug trade name, original title, device manufacturer, drug manufacturer, device trade name, keyword, floating subheading word, candidate term word] (11)

35 exp non insulin dependent diabetes mellitus/ (219348)

36 type II diabet*.mp. [mp=title, abstract, heading word, drug trade name, original title, device manufacturer, drug manufacturer, device trade name, keyword, floating subheading word, candidate term word] (13830)

37 type 2 diabet*.mp. [mp=title, abstract, heading word, drug trade name, original title, device manufacturer, drug manufacturer, device trade name, keyword, floating subheading word, candidate term word] (176293)

38 35 or 36 or 37 (261007)

39 ertugliflozin.mp. [mp=title, abstract, heading word, drug trade name, original title, device manufacturer, drug manufacturer, device trade name, keyword, floating subheading word, candidate term word] (200)

40 exp ertugliflozin/ (196)

41 segluromet.mp. [mp=title, abstract, heading word, drug trade name, original title, device manufacturer, drug manufacturer, device trade name, keyword, floating subheading word, candidate term word] (0)

42 stelagtro.mp. [mp=title, abstract, heading word, drug trade name, original title, device manufacturer, drug manufacturer, device trade name, keyword, floating subheading word, candidate term word] (0)

43 1 or 2 or 3 or 4 or 5 or 6 or 7 or 8 or 9 or 10 or 11 or 12 or 13 or 14 or 15 or 16 or 17 or 18 or 19 or 20 or 21 or 22 or 23 or 24 or 25 or 26 or 27 or 28 or 29 or 30 or 31 or 32 or 33 or 34 or 39 or 40 or 41 or 42 (7119)

44 38 and 43 (5329)

45 (systematic review or meta-analys*).mp. [mp=title, abstract, heading word, drug trade name, original title, device manufacturer, drug manufacturer, device trade name, keyword, floating subheading word, candidate term word] (366577)

46 44 and 45 (613)

**Cochrane Library Search Strategy**

([mh "sodium glucose transporter 2"] OR sodium-glucose co-transporter 2 inhibitor* OR SGLT-2 inhibitor* OR sodium glucose linked transporter 2 inhibitor OR canagliflozin OR [mh "canagliflozin"] Invokana OR Invokamet OR dapagliflozin OR Forxiga OR Xigduo OR empagliflozin OR Jardiance OR Synjardy OR ertugliflozin OR Stelagtro OR Segluromet OR atigliflozin OR bexagliflozin OR ipragliflozin OR Suglat OR luseogliflozin OR Lusefi OR remogliflozin OR Sergliflozin OR tofogliflozin OR Apleway OR Deberza)

AND

([mh "diabetes mellitus, type 2"] OR type 2 diabet* OR type II diabet*)

Limit to Cochrane Reviews

Table S1. Excluded studies and reasons for exclusion.

| **Author and Year** | **Reason for Exclusion** |
| --- | --- |
| Azharuddin 2018^1^ | No cancer events reported. |
| Baker 2014^2^ | No cancer events reported. |
| Barnett 2016^3^ | SGLT2 inhibitors not main drug class of interest. |
| Berhan 2013^4^ | No cancer events reported. |
| Bundhun 2017^5^ | No cancer events reported. |
| Bundhun 2018^6^ | SGLT2 inhibitors not main drug class of interest. |
| Cahyadi 2018^7^ | No cancer events reported. |
| Cai 2018^8^ | No cancer events reported. |
| Cai 2017^9^ | SGLT2 inhibitors not main drug class of interest. |
| Cai 2018^10^ | No safety outcome addressed beyond hypoglycemia. |
| Cheng 2019^11^ | No cancer events reported. |
| Cho 2018^12^ | No cancer events reported. |
| Cho 2018^13^ | SGLT2 inhibitors not main drug class of interest. |
| Cintra 2019^14^ | No cancer events reported. |
| Clar 2012^15^ | No cancer events reported. |
| Devi 2017^16^ | No cancer events reported. |
| Donnan 2018^17^ | No cancer events reported. |
| Donnan 2019^18^ | No cancer events reported. |
| Downes 2015^19^ | SGLT2 inhibitors not main drug class of interest. |
| Egger 2016^20^ | SGLT2 inhibitors not main drug class of interest. |
| Elgebaly 2018^21^ | No cancer events reported. |
| Fadini 2018^22^ | No cancer events reported. |
| Fan 2016^23^ | No cancer events reported. |
| Fei 2018^24^ | SGLT2 inhibitors not main drug class of interest. |
| Feng 2019^25^ | No safety outcome addressed beyond hypoglycemia. |
| Gilbert 2019^26^ | Not a systematic review. |
| Goring 2014^27^ | No safety outcome addressed beyond hypoglycemia. |
| Guo 2018^28^ | No cancer events reported. |
| Hemmingsen 2016^29^ | No quantitative synthesis of results. |
| Hinnen 2015^30^ | Not a systematic review. |
| Hussein 2019^31^ | SGLT2 inhibitors not main drug class of interest. |
| Imprialos 2017^32^ | Not a systematic review. |
| Inoue 2019^33^ | SGLT2 inhibitors not main drug class of interest. |
| Jabbour 2018^34^ | Not a systematic review. |
| Johnsson 2013^35^ | Not a systematic review. |
| Johnston 2017^36^ | No quantitative synthesis of results. |
| Kaur 2015^37^ | No cancer events reported. |
| Kawalec 2014^38^ | SGLT2 inhibitors not main drug class of interest. |
| Kohler 2016^39^ | Not a systematic review. |
| Kohler 2018^40^ | Not a systematic review. |
| Koye 2017^41^ | SGLT2 inhibitors not main drug class of interest. |
| Kramer 2018^42^ | SGLT2 inhibitors not main drug class of interest. |
| Li 2017^43^ | No cancer events reported. |
| Li 2017^44^ | No cancer events reported. |
| Li 2018^45^ | No cancer events reported. |
| Li 2018^46^ | No cancer events reported. |
| Li 2018^47^ | No cancer events reported. |
| Li 2019^48^ | No cancer events reported. |
| Liakos 2014^49^ | No cancer events reported. |
| Liao 2018^50^ | No cancer events reported. |
| Liu 2015^51^ | No cancer events reported. |
| Liu 2017^52^ | No cancer events reported. |
| Lorenzi 2017^53^ | SGLT2 inhibitors not main drug class of interest. |
| Maruthur 2016^54^ | SGLT2 inhibitors not main drug class of interest. |
| Mazidi 2017^55^ | No cancer events reported. |
| McNeill 2019^56^ | No cancer events reported. |
| Mearns 2015^57^ | SGLT2 inhibitors not main drug class of interest. |
| Meng 2016^58^ | No safety outcome addressed beyond hypoglycemia. |
| Milder 2019^59^ | No cancer events reported. |
| Mishriky 2018^60^ | No cancer events reported. |
| Monami 2017^61^ | No cancer events reported. |
| Monami 2017^62^ | No safety outcome addressed beyond hypoglycemia. |
| Monami 2017^63^ | Not a systematic review. |
| Monami 2018^64^ | No safety outcome addressed beyond hypoglycemia. |
| Musso 2012^65^ | No cancer events reported. |
| Orme 2013^66^ | Conference abstracts. |
| Parveen 2016^67^ | No quantitative synthesis of results. |
| Puckrin 2018^68^ | No cancer events reported. |
| Qian 2018^69^ | SGLT2 inhibitors not main drug class of interest. |
| Ruanpeng 2017^70^ | No cancer events reported. |
| Saad 2017^71^ | No cancer events reported. |
| Salsali 2017^72^ | Not a systematic review. |
| Savarese 2016^73^ | SGLT2 inhibitors not main drug class of interest. |
| Seidu 2018^74^ | No safety outcome addressed beyond hypoglycemia. |
| Shyangdan 2016^75^ | No safety outcome addressed beyond hypoglycemia. |
| Singh 2019^76^ | No safety outcome addressed beyond hypoglycemia. |
| Sonesson 2016^77^ | Not a systematic review. |
| Sun 2014^78^ | No safety outcome addressed beyond hypoglycemia. |
| Tang 2016^79^ | No cancer events reported. |
| Tang 2016^80^ | No cancer events reported. |
| Tang 2016^81^ | No cancer events reported. |
| Tang 2017^82^ | No cancer events reported. |
| Tang 2017^83^ | No cancer events reported. |
| Tang 2016^84^ | No safety outcome addressed beyond hypoglycemia. |
| Toyama 2019^85^ | No cancer events reported. |
| Usman 2018^86^ | No safety outcome addressed beyond hypoglycemia. |
| Vasilakou 2013^87^ | No cancer events reported. |
| Wang 2016^88^ | No cancer events reported. |
| Wang 2018^89^ | No cancer events reported. |
| Wang 2019^90^ | No cancer events reported. |
| Wang 2018^91^ | SGLT2 inhibitors not main drug class of interest. |
| Wang 2018^92^ | Not English. |
| Wang 2019^93^ | No safety outcome addressed beyond hypoglycemia. |
| Willis 2019^94^ | SGLT2 inhibitors not main drug class of interest. |
| Wu 2019^95^ | No cancer events reported. |
| Xiong 2016^96^ | No cancer events reported. |
| Xu 2017^97^ | No cancer events reported. |
| Yamani 2019^98^ | No safety outcome addressed beyond hypoglycemia. |
| Yang 2014^99^ | No cancer events reported. |
| Yang 2015^100^ | No cancer events reported. |
| Yang 2017^101^ | No cancer events reported. |
| Zaccardi 2016^102^ | No cancer events reported. |
| Zelniker 2019^103^ | No cancer events reported. |
| Zhang 2014^104^ | No cancer events reported. |
| Zhang 2018^105^ | No cancer events reported. |
| Zhang 2018^106^ | No cancer events reported. |
| Zhang 2018^107^ | No cancer events reported. |
| Zhang 2014^108^ | No safety outcome addressed beyond hypoglycemia. |
| Zhao 2018^109^ | No safety outcome addressed beyond hypoglycemia. |
| Zhao 2019^110^ | No safety outcome addressed beyond hypoglycemia. |
| Zheng 2018^111^ | SGLT2 inhibitors not main drug class of interest. |
| Zhong 2016^112^ | No cancer events reported. |
| Zhong 2016^113^ | No cancer events reported. |

**References of Excluded Studies**

1. Azharuddin M, Adil M, Ghosh P, Sharma M. Sodium-glucose cotransporter 2 inhibitors and fracture risk in patients with type 2 diabetes mellitus: A systematic literature review and Bayesian network meta-analysis of randomized controlled trials. *Diabetes Res Clin Pract*. 2018;146:180-190. doi: 10.1016/j.diabres.2018.10.019.
2. Baker WL, Symth LR, Bourret EM, Chamberlin KW, White WB. Effects of sodium-glucose co-transporter 2 inhibitors on blood pressure: a systematic review and meta-analysis. *J AM Soc Hypertens*. 2014;8(4):262-275. doi: 10.1016/j.jash.2014.01.007.
3. Barnett AH, Orme ME, Fenici P, Townsend R, Wygant G, Roudaut M. Systematic Review and Network Meta-Analysis to Compare Dapagliflozin with other Diabetes Medications in Combination with Metformin for Adults with Type 2 Diabetes. *Intern Med*. 2014;S6:006. doi: 10.4172/2165-8048.S6-006.
4. Berhan A, Barker A. Sodium glucose co-transport 2 inhibitors in the treatment of type 2 diabetes mellitus: a meta-analysis of randomized double-blind controlled trials. *BMC Endocr Disord*. 2013. 13:58. doi: 10.1186/1472-6823-13-58.
5. Bundhun PK, Janoo G, Huang F. Adverse drug events observed in patients with type 2 diabetes mellitus treated with 100 mg versus 300 mg canagliflozin: a systematic review and meta-analysis of published randomized controlled trials. *BMC Pharmacol Toxicol*. 2017;18(1):19. doi: 10.1186/s40360-017-0126-9.
6. Bundhun PK, Huang F. Adverse Drug Events Associated with sitagliptin Versus canagliflozin for the Treatment of Patients with Type 2 Diabetes Mellitus: A Systematic Comparison Through a Meta-Analysis. *Diabetes Ther.* 2018;9:1883-1895. doi: 10.1007/s13300-018-0481-6.
7. Cahyadi A, Jimeno CA. Blood Pressure Lowering Effects of Sodium Glucose Transporter 2 Inhibitors Among Adult Patients with Type 2 Diabetes Mellitus: A Meta-Analysis. *PJIM*. 2018;56(3):176-188.
8. Cai X, Gao X, Yang W, et al. No disparity of the efficacy and all-cause mortality between Asian and non-Asian type 2 diabetes patients with sodium-glucose cotransporter 2 inhibitors treatment: A meta-analysis. *J Diabetes Investig*. 2018;9(4):850-861. doi: 10.1111/jdi.12760.
9. Cai X, Ji l, Chen Y, et al. Comparisons of weight changes between sodium-glucose cotransporter 2 inhibitors treatment and glucagon-like peptide-1 analogs treatment in type 2 diabetes patients: A meta-analysis. *J Diabetes Investig*. 2017;8:510-517. doi: 10.1111/jdi.12625.
10. Cai X, Yang W, Gao X, et al. The Association Between the Dosage of SGLT2 Inhibitor and Weight Reduction in Type 2 Diabetes Patients: A Meta-Analysis. *Obesity*. 2018;26:70-80. doi: 10.1002/oby.22066.
11. Cheng L, Li YY, Hu W, et al. Risk of bone fracture associated with sodium-glucose cotransporter-2 inhibitor treatment: A meta-analysis of randomized controlled trials. *Diabetes Metab*. 2019;pii:S1262-3636(19)30025-4. doi: 10.1016/j.diabet.2019.01.010.
12. Cho YK, Kang YM, Lee SE, et al. Efficacy and safety of combination therapy with SGLT2 and DPP4 inhibitors in the treatment of type 2 diabetes: A systematic review and meta-analysis. *Diabetes Metab*. 2018;44(5):393-401. doi: 10.1016/j.diabet.2018.01.011.
13. Cho YK, Kim YJ, Kang YM, et al. Comparison between sodium-glucose cotransporter 2 inhibitors and pioglitazone as additions to insulin therapy in type 2 diabetes patients: A systematic review with an indirect comparison meta-analysis. *J Diabetes Investig*. 2018;9:882-892. doi: 10.1111/jdi.12787.
14. Cintra R, Moura FA, Carvalho LSF, et al. Inhibition of the sodium-glucose co-transporter 2 in the elderly: clinical and mechanistic insights into safety and efficacy. *Rev Assoc Med Bras*. 2019;65(1):70-86. doi: 10.1590/1806-9282.65.1.70.
15. Clar C, Gill GA, Court R, Waugh N. Systematic review of SGLT2 receptor inhibitors in dual or triple therapy in type 2 diabetes. *BMJ Open*. 2012;2:e001007. doi: 10.1136/bmjopen-2012-001007.
16. Devi R, Mali G, Chakraborty I, Unnikrishnan MK, Abdulsalim S. Efficacy and safety of empagliflozin in type 2 diabetes mellitus: a meta-analysis of randomized controlled trials. *Postgrad Med*. 2017;129(3):382-392. doi: 10.1080/00325481/2017/1259544.
17. Donnan JR, Grandy CA, Chibrikov E, et al. Dose response of sodium glucose cotransporter-2 inhibitors in relation to urinary tract infections: a systematic review and network meta-analysis of randomized controlled trials. *CMAJ Open*. 2018;6(4):E594-E602. doi: 10.9778/cmajo.20180111.
18. Donnan JR, Grandy CA, Marra CA et al. Comparative safety of the sodium glucose co-transporter 2 (SGLT2) inhibitors: a systematic review and meta-analysis. *BMJ Open*. 2019;9(1):e022577. doi: 10.1136/bmjopen-2018-022577.
19. Downes MJ, Bettington EK, Gunton JE, Turkstra E. Triple therapy in type 2 diabetes; a systematic review and network meta-analysis. *PeerJ*. 2015;3:e1461. doi: 10.7717/peerj.1461.
20. Egger A, Kraenzlin ME, Meier C. Effects of Incretin-Based Therapies and SGLT2 Inhibitors on Skeletal Health. *Curr Osteoporos Rep*. 2016;14:345-350. doi: 10.1007/s11914-016-0337-9.
21. Elgebaly A, Abdelazeim M, Abdelazeim B, et al. Tolerability and Efficacy of Ipragliflozin in The Management of Inadequately Controlled Type 2 Diabetes mellitus: A Systematic Review and Meta-Analysis. *Exp Clin Endocrinol Diabetes*. 2018. doi: 10.1055/a-0579-7860.
22. Fadini GP, Bonora BM, Mayur S, Rigato M, Avogaro A. Dipeptidyl peptidase-4 inhibitors moderate the risk of genitourinary tract infections associated with sodium-glucose co-transporter 2 inhibitors. *Diabetes Obes Metab*. 2018;20(3):740-744. doi: 10.1111/dom.13130.
23. Fan G, Han R, Zhang Y, et al. Safety assessment of canagliflozin for type 2 diabetes mellitus. *Int J Clin Med*. 2016;9(2):2595-2612.
24. Fei Y, Tsoi MF, Kumana CR, Cheung TS, Cheung BMY. Network meta-analysis of cardiovascular outcomes in randomized controlled trials of new antidiabetic drugs. *Int J Cardiol*. 2018;254:291-296. doi: 10.1016/j.ijcard.2017/12/039.
25. Feng C, Wu M, Chen Z, et al. Effect of SGLT2 inhibitors on renal function in patients with type 2 diabetes mellitus: a systematic review and meta-analysis of randomized controlled trials. *Int Urol Nephrol*. 2019;51(4):655-669. doi: 10.1007/s11255-019-02112-6.
26. Gibert RE, Thorpe KE. Acute kidney injury with sodium-glucose co-transporter 2 inhibitors: A meta-analysis of cardiovascular outcome trials. *Diabetes Obes Metab*. 2019;21(8):1996-2000. doi: 10.1111/dom.13754.
27. Goring S, Hawkins N, Wygant G, et al. Dapagliflozin compared with other oral anti-diabetes treatments when added to metformin monotherapy: a systematic review and network meta-analysis. *Diabetes Obes Metab*. 2014;16(5):433-442. doi: 10.1111/dom.12239.
28. Guo M, Ding J, Li J, et al. SGLT2 inhibitors and risk of stroke in patients with type 2 diabetes: A systematic review and meta-analysis. *Diabetes Obes Metab*. 2018;20(8):1977-1982. doi: 10.1111/dom.13295.
29. Hemmingsen B, Krogh J, Metzendorf MI, Richter B. Sodium-glucose cotransporter (SGLT) 2 inhibitors for prevention or delay of type 2 diabetes and its associated complications in people at risk for the development of type 2 diabetes mellitus. *Cochrane Database of Syst Rev*. 2016;4:CD012106. doi: 10.1002/14651858.CD012106.pub2.
30. Hinnen D. Glucuretic effects and renal safety of dapagliflozin in patients with type 2 diabetes. *Ther Adv Endocrinol Metab*. 2015;6(3):92-102. doi: 10.1177/2042019915575273.
31. Hussein H, Zaccardi F, Khunti K, Seidu S, Davies MJ, Gray LJ. Cardiovascular efficacy and safety of sodium-glucose co-transporter-2 inhibitors and glucagon-like peptide-1 receptor agonists: a systematic review and network meta-analysis. *Diabet Med*. 2019;36:444-452. doi: 10.1111/dme.13898.
32. Imprialos K, Faselis C, Boutari C, et al. SGLT-2 Inhibitors and Cardiovascular Risk in Diabetes Mellitus: A Comprehensive and Critical Review of the Literature. *Curr Pharm Des*. 2017;23(10):1510-1521. doi: 10.2174/1381612823666170124123927.
33. Inoue H, Tamaki Y, Kashihara Y, et al. Efficacy of DPP-4 inhibitors, GLP-1 analogues, and SGLT2 inhibitors as add-ons to metformin monotherapy in T2DM patients: a model based meta-analysis. *Br J Clin Pharmacol*. 2019;85:393-402. doi: 10.1111/bcp.13807.
34. Jabbour S, Seufert J, Scheen A, Bailey CJ, Karup C, Langkilde AM. Dapagliflozin in patients with type 2 diabetes mellitus: A pooled analysis of safety data from phase IIb/III clinical trials. *Diabetes Obes Metab*. 2018;20:620-628. doi: 10.1111/dom.13124.
35. Johnsson KM, Ptaszynska A, Schmitz B, Sugg J, Parikh SJ, List JF. Urinary tract infections in patients with diabetes treated with dapagliflozin. *J Diabetes Complications*. 2013;27(5):473-478. doi: 10.1016/j.jdiacomp.2013.05.004.
36. Johnston R, Uthman O, Cummins E, et al. Canagliflozin, dapagliflozin and empagliflozin monotherapy for treating type 2 diabetes: systematic review and economic evaluation. *Health Technol Assess*. 2017;21(2):1-218. doi: 10.3310/hta21020.
37. Kaur K, Likar N, Dang A, Kaur G. Efficacy and safety of canagliflozin among patients with type 2 diabetes mellitus: A systematic review and meta-analysis. *Indian J Endocrinol Metab*. 2015;19(6):705-721. doi: 10.4103/2230-8210.167562.
38. Kawalec P, Mikrut A, Lopuch S. The safety of dipeptidyl peptidase-4 (DPP-4) inhibitors or sodium-glucose cotransporter 2 (SLGT-2) inhibitors added to metformin background therapy in patients with type 2 diabetes mellitus: a systematic review and meta-analysis. *Diabetes Metab Res Rev*. 2014;30:269-283. doi: 10.1002/dmrr.2494.
39. Kohler S, Kaspers S, Salsali A, Zeller C, Woerle HJ. Analysis of Fractures in Patients With Type 2 Diabetes Treated With Empagliflozin in Pooled Data From Placebo-Controlled Trials and a Head-to-Head Study Versus Glimepiride. *Diabetes Care*. 2018;41(8):1809-1816. doi: 10.2337/dc17-1525.
40. Kohler S, Salsali A, Hantel S, et al. Safety and Tolerability of Empagliflozin in Patients with Type 2 Diabetes. *Clin Ther*. 2016;28(6):1299-1313. doi: 10.1016/j.clinthera.2016.03.031.
41. Koye DN, Shaw JE, Reid CM, Atkins RC, Reutens AT, Magliano DJ. Incidence of chronic kidney disease among people with diabetes: a systematic review of observational studies. *Diabet Med*. 2017;34:887-901. doi: 10.1111/dme.13324.
42. Kramer CK, Ye C, Campbell S, Retnakaran R. Comparison of New Glucose-Lowering Drugs on Risk of Heart Failure in Type 2 Diabetes: A Network Meta-Analysis. *JACC Heart Fail*. 2018;6(10):823-830. doi: 10.1016/j.jchf.2018.05.021.
43. Li D, Wang T, Shen S, Fang Z, Dong Y, Tang H. Urinary tract and genital infections in patients with type 2 diabetes treated with sodium-glucose co-transporter 2 inhibitors: A meta-analysis of randomized controlled trials. *Diabetes Obes Metab*. 2017;19(3):348-355. doi: 10.1111/dom.12825.
44. Li J, Gong Y, Li C, Lu Y, Liu Y, Shao Y. Long-term efficacy and safety of sodium-glucose cotransporter-2 inhibitors as add-on to metformin treatment in the management of type 2 diabetes mellitus: A meta-analysis. *Medicine*. 2017;96(27):se7201. doi: 10.1097/MD.0000000000007201.
45. Li D, Shi W, Wang T, Tang H. SGLT2 inhibitor plus DPP-4 inhibitor as combination therapy for type 2 diabetes: A systematic review and meta-analysis. *Diabetes Obes Metab*. 2018;20(8):1972-1976. doi: 10.1111/dom.13294.
46. Li D, Yang JY, Wang T, Shen S, Tang H. Risks of diabetic foot syndrome and amputation associated with sodium glucose co-transporter 2 inhibitors: A meta-analysis of Randomized Controlled Trials. *Diabetes Metab*. 2018;44(5):410-414. doi: 10.1016/j.diabet.2018.02.001.
47. Li J, Shao YH, Wang XG, Gong Y, Li C, Lu Y. Efficacy and safety of sodium-glucose contransporter 2 inhibitors as add-on to metformin and sulfonylurea treatment for the management of type 2 diabetes: a meta-analysis. *Endocr J*. 2018;65(3):335-344. doi: 10.1507/endocrj.EJ17-0372.
48. Li X, Ting L, Cheng Y, et al. Effects of SGLT2 inhibitors on fractures and bone mineral density in type 2 diabetes: An updated meta-analysis. *Diabetes Metab Res Rev*. 2019;e3170. doi: 10.1002/dmrr.3170.
49. Liakos A, Karagiannis T, Athanasiadou E, et al. Efficacy and safety of empagliflozin for type 2 diabetes: a systematic review and meta-analysis. *Diabetes Obes Metab*. 2014;16(10):984-993. doi: 10.1111/dom.12307.
50. Liao HW, Wu YL, Sue YM, Lee M, Ovbiagele B. Sodium-glucose contransporter 2 inhibitor plus pioglitazone vs. pioglitazone alone in patients with diabetes mellitus: A systematic review and meta-analysis of randomized controlled trials. *Endocrinol Diabetes Metab*. 2018;2(1):e00050. doi: 10.1002edm2.50.
51. Liu XY, Zhang N, Chen R, Zhao JG, Yu P. Efficacy and safety of sodium-glucose contransporter 2 inhibitors in type 2 diabetes: a meta0analysis of randomized controlled trials for 1 to 2 years. *J Diabetes Complications*. 2015;29(8):1295-1303. doi: 10.1016/j.jdiacomp.2015.07.011.
52. Liu J, Li L, Li S, et al. Effects of SGLT2 inhibitors on UTIs and genital infections in type 2 diabetes mellitus: a systematic review and meta-analysis. *Sci Rep*. 2017;7(1):2824. doi: 10.1038/s41598-017-02733-w.
53. Lorenzi M, Ploug UJ, Langer J, Skovgaard R, Zoratti M, Jansen J. Liraglutide Versus SGLT2-Inhibitors in People with Type 2 Diabetes: A Network Meta-Analysis. *Diabetes Ther*. 2017;8:85-99. doi: 10.1007/s13300-016-0217-4.
54. Marthur NM, Tseng E, Hutfless S, et al. Diabetes Medications as Monotherapy or Metformin-Based Combination Therapy for Type 2 Diabetes: A Systematic Review and Meta-analysis. *Ann Intern Med*. 2016;164(11):740-751. doi: 10.7326/M15-2650.
55. Mazidi M, Rezaie P, Gao HK, Kengne AP. Effect of Sodium-Glucose Cotransport-2 Inhibitors on Blood Pressure in People With Type 2 Diabetes Mellitus: A Systematic Review and Meta-Analysis of 43 Randomized Control Trials With 22528 Patients. *J Am Heart Assoc*. 2017;6(6):e004007. doi: 10.1161/JAHA.116.004007.
56. McNeill AM, Davies G, Kruger E, et al. Ertugliflozin Compared to Other Anti-hyperglycemic Agents as Monotherapy and Add-on Therapy in Type 2 Diabetes: A Systematic Literature Review and Network Meta-Analysis. *Diabetes Ther*. 2019;10(2):473-491. doi: 10.1007/s13300-019-0566-x.
57. Mearns ES, Sobieraj DM, White CM, et al. Comparative Efficacy and Safety of Antidiatice Drug Regimens Added to Metformin Monotherapy in Patients with Type 2 Diabetes: A Network Meta-Analysis. *PLoS ONE*. 2015;10(4):e0125879. doi: 10.1371/journal.pone.0125879.
58. Meng Q, Shen Y, Liu D, Jiang F. Efficacy of canagliflozin combined with antidiabetic drugs in treating type 2 diabetes mellitus: Meta-analysis of randomized control trials. *J Diabetes Investig*. 2016;7:359-365. doi: 10.1111/jdi.12417.
59. Milder TY, Stocker SL, Abdel Shaheed C, et al. Combination Therapy with an SGLT2 Inhibitor as Initial Treatment for Type 2 Diabetes: A Systematic Review and Meta-Analysis. *J Clin Med*. 2019;8(1):E45. doi: 10.3390/jcm8010045.
60. Mishriky BM, Tanenberg RJ, Sewell KA, Cummings DM. Comparing SGLT-2 inhibitors to DPP-4 inhibitors as an add-on therapy to metformin in patients with type 2 diabetes: A systematic review and meta-analysis. *Diabetes Metab*. 2018;44(2):112-120. doi: 10.1016/j.diabet.2018.01.017.
61. Monami M, Nreu B, Zannoni S, Lualdi C, Mannucci E. Effects of SLGT-2 inhibitors on diabetic ketoacidosis: A meta-analysis of randomised controlled trials. *Diabetes Red Clin Pract*. 2017;130:53-60. doi: 10.1016/j.diabres/2017.04.017.
62. Monami M, Dicembrini I, Mannucci E. Effects of SGLT-2 inhibitors on mortality and cardiovascular events: a comprehensive meta-analysis of randomized controlled trials. *Acta Diabetol*. 2017;54:19-36. doi: 10.1007/s00592-016-0892-7.
63. Monami M, Zannoni S, Nreu B, Mannucci E. Toe amputations with SGLT-2 inhibitors: data from randomized clinical trials. *Acta Diabetol*. 2017;54:411-413. doi: 10.1007/s00592-016-0928-z.
64. Monami M, Liistro F, Scatena A, Nreu B, Mannucci E. Short and medium-term efficacy of sodium glucose co-transporter-2 (SGLT-2) inhibitors: A meta-analysis of randomized controlled trials. *Diabetes Obes Metab*. 2018;20:1213-1222. doi: 10.1111/dom/13221.
65. Musso G, Gambino R, Cassader M, Pagano G. A novel approach to control hyperglycemia in type 2 diabetes: sodium glucose co-transport (SGLT) inhibitors: systematic review and meta-analysis of randomized trials. *Ann Med*. 2012;44(4):375-393. doi: 10.3109/07853890.2011.560181.
66. Orme ME, Fenici P, Lomon ID, Wygant G, Townsend R, Roudaut M. A Systematic Review and Network Meta-Analysis of Second-Line Anti-Diabetes Treatments for Those with Type 2 Diabetes Mellitus Inadequately Controlled by Sulfonylurea Monotherapy. *Value Health*. 2013;16(7):A432. doi: 10.1016/j.jval.2013.08.629.
67. Parveen R, Agarwal NB, Kaushal N, Mali G, Raisuddin S. Efficacy and safety of canagliflozin in type 2 diabetes mellitus: systematic review of randomized controlled trials. *Expert Opin Pharmacother*. 2016;17(1):105-115. doi: 10.1517/14656566.2016.1109629.
68. Puckrin R, Saltiel MP, Reynier P, Azoulay L, Yu OHY, Filion KB. SGLT-2 inhibitors and the risk of infections: a systematic review and meta-analysis of randomized controlled trials. *Acta Diabetol*. 2018;55(5):503-514. doi: 10.1007/s00592-018-1116-0.
69. Qian D, Zhang T, Tan X, et al. Comparison of antidiabetic drugs added to sulfonylurea monotherapy in patients with type 2 diabetes mellitus: A network meta-analysis. 2018;13(8):e0202563. doi: 10.1371/journal.pone.0202563.
70. Ruanpeng D, Ungprasert P, Sangtian J, Harindhanavudhi T. Sodium-glucose cotransporter 2 (SGLT2) inhibitors and fracture risk in patients with type 2 diabetes mellitus: A meta-analysis. *Diabetes Metab Res Rev*. 2017;33(6). doi: 10.1002/dmrr.2903.
71. Saad M, Mahmoud AN, Elgendy IY, et al. Cardiovascular outcomes with sodium-glucose contransporter-2 inhibitors in patients with type II diabetes mellitus: A meta-analysis of placebo-controlled randomized trials. *Int J Cardiol*. 2017;228:352-358. doi: 10.1016/j.ijcard.2016.11.181.
72. Salsali A, Kim G, Woerle HJ, Broedl UC, Hantel S. Cardiovascular safety of empagliflozin in patients with type 2 diabetes: a meta-analysis of data from randomized placebo-controlled trials. *Diabetes Obes Metab*. 2016;18(10):1034-1040. doi: 10.1111/dom.12734.
73. Savarese G, D’Amore C, Federici M, et al. Effects of Dipeptidyl Peptidase 4 Inhibitors and Sodium-Glucose Linked cotransporter-2 Inhibitors on cardiovascular events in patients with type 2 diabetes mellitus: A meta-analysis. *Int J Cardiol*. 2016;220:595-601. doi: 10.1016/sj.ijcard.2016.06.208.
74. Seidu S, Kunutsor SK, Cos X, Gillani S, Khunti K. SGLT2 inhibitors and renal outcomes in type 2 diabetes with or without renal impairment: A systematic review and meta-analysis. *Prim Care Diabetes*. 2018;12(3):265-283. doi: 10.1016/j.pcd.2018.02.001.
75. Shyangdan DS, Uthman OA, Waugh N. SGLT-2 receptor inhibitors for treating patients with type 2 diabetes mellitus: a systematic review and network meta-analysis. *BMJ Open*. 2016;6:e009417. doi: 10.1136/bmjopen-2015-009417.
76. Singh AK, Singh R. Heart failure hospitalization with SGLT-2 inhibitors: a systematic review and meta-analysis of randomized controlled and observational studies. *Expert Rev Clin Pharmacol*. 2019;12(4):299-308. doi: 10.1080/17512433.2019.1588110.
77. Sonesson C, Johansson PA, Johnsson E, Gause-Nilsson I. Cardiovascular effects of dapagliflozin in patients with type 2 diabetes and different risk categories: a meta-analysis. *Cardiovasc Diabetol*. 2016;15:37. doi: 10.1186/s12933-016-0356-y.
78. Sun Y, Zhou Y, Chen X, Che W, Leung S. The efficacy of dapagliflozin combined with hypoglycemic drugs in treating type 2 diabetes mellitus: meta-analysis of randomised controlled trials. *BMJ Open*. 2014;4:e004619. doi: 10.1136/bmjopen-2013-004619.
79. Tang H, Li D, Wang T, Zhai S, Song Y. Effect of Sodium-Glucose Cotransporter 2 Inhibitors on Diabetic Ketoacidosis Among Patients With Type 2 Diabetes: A Meta-analysis of Randomized Controlled Trials. *Diabetes Care*. 2016; 39(8):e123-124. Doi: 10.2337/dc16-0885.
80. Tang H, Zhang X, Zhang J, et al. Elevated serum magnesium associated with SGLT2 inhibitor use in type 2 diabetes patients: a meta-analysis of randomised controlled trials. *Diabetologia*. 2016;59:2546-2551. doi: 10.1007/s00125-016-4101-6.
81. Tang HL, Li DD, Zhang JJ, et al. Lack of evidence for a harmful effect of sodium-glucose co-transporter 2 (SGLT2) inhibitors on fracture risk among type 2 diabetes patients: a network and cumulative meta-analysis of randomized controlled trials. *Diabetes Obes Metab*. 2016;18(12):1199-1206. doi: 10.1111/dom.12742.
82. Tang H, Cui W, Li D, et al. Sodium-glucose co-transporter 2 inhibitors in addition to insulin therapy for management of type 2 diabetes mellitus: A meta-analysis of randomized controlled trials. *Diabetes Obes Metab*. 2017;19(1):142-147. doi: 10.1111/dom.12785.
83. Tang H, Li D, Zhang J, et al. Sodium-glucose co-transporter-2 inhibitors and risk of adverse renal outcomes among patients with type 2 diabetes: A network and cumulative meta-analysis of randomized controlled trials. *Diabetes Obes Metab*. 2017;19(8):1106-1115. doi: 10.1111/dom.12917.
84. Tang H, Fang Z, Wang T, Cui W, Zhai S, Song Y. Meta-Analysis of Effects of Sodium-Glucose Cotransporter 2 Inhibitors on Cardiovascular Outcomes and All-Cause Mortality Among Patients With Type 2 Diabetes Mellitus. *Am J Cardiol*. 2016;118(11):1774-1780. doi: 10.1016/j/amjcard.2016.08.061.
85. Toyama T, Neuen BL, Jun M, et al. Effect of SGLT2 inhibitors on cardiovascular, renal and safety outcomes in patients with type 2 diabetes mellitus and chronic kidney disease: A systematic review and meta-analysis. *Diabetes Obes Metab*. 2019;21(5):1237-1250. doi: 10.1111/dom.13648.
86. Usman MS, Siddigi TJ, Memon MM, et al. Sodium-glucose co-transporter 2 inhibitors and cardiovascular outcomes: A systematic review and meta-analysis. *Eur J Prev Cardiol*. 2018;25(5):495-502. doi: 10.1177/2047487318755531.
87. Vasilakou D, Karagiannis T, Athanasiadou E, et al. Sodium-glucose cotransporter 2 inhibitors for type 2 diabetes: a systematic review and meta-analysis. *Ann Intern Med*. 2013;159(4):262-274. doi: 10.7326/0003-4819-159-4-201308200-00007.
88. Wang Y, Hu X, Liu X, Wang Z. An overview of the effect of sodium glucose cotransporter 2 inhibitor monotherapy on glycemic and other clinical laboratory parameters in type 2 diabetes patients. *Ther Clin Risk Manag*. 2016;12:1113-1131. doi: 10.2147/TCRM.S112236.
89. Wang Z, Sun J, Han R, et al. Efficacy and safety of sodium-glucose cotransporter-2 inhibitors versus dipeptidyl peptidase-4 inhibitors as monotherapy or add-on to metformin in patients with type 2 diabetes mellitus: A systematic review and meta-analysis. *Diabetes Obes Metab*. 2018;20(1):113-120. doi: 10.1111/dom.13047.
90. Wang C, Zhou Y, Kong Z, et al. The renoprotective effects of sodium-glucose cotransporter 2 inhibitors versus placebo in patients with type 2 diabetes with or without prevalent kidney disease: A systematic review and meta-analysis. *Diabetes Obes Metab*. 2019;21(4):1018-1026. doi: 10.1111/dom.13620.
91. Wang K, Zhang Y, Zhao C, Jiang M. SGLT-2 Inhibitors and DPP-4 Inhibitors as Second-Line Drugs in Patients with Type 2 Diabetes: A Meta-Analysis of Randomized Clinical Trials. *Horm Metab Res*. 2018;50(10):768-777. doi: 10.1055/a-0733-7919.
92. Wang ZY, Sun JW, Li KB, Yang H. SGLT2 inhibitors for T2DM patients with moderate renal impairment: a meta-analysis. *Chinese Journal of New Drugs*. 2018;27(22):2693-2699.
93. Wang H, Yang J, Chen X, Qiu F, Li J. Effects of sodium-glucose Cotransporter 2 Inhibitor Monotherapy on Weight Changes in Patients With Type 2 Diabetes Mellitus: A Bayesian Network Meta-analysis. *Clin Ther*. 2019;41(2):322-334.e11. doi: 10.1016/j.clinthera.2019.01.001.
94. Willis M, Asseburg C, Neslusan C. Conducting and interpreting results of network meta-analyses in type 2 diabetes mellitus: A review of network meta-analyses that include sodium-glucose co-transporter 2 inhibitors. *Diabetes Res Clin Pract*. 2019;148:222-233. doi: 10.1016/j.diabres.2019.01.005.
95. Wu B, Zheng H, Gu J, et al. Effects of sodium-glucose cotransporter 2 inhibitors in addition to insulin therapy on cardiovascular risk factors in type 2 diabetes patients: A meta-analysis of randomized controlled trials. *J Diabetes Investig*. 2019;10(2):446-457. doi: 10.1111/jdi.12876.
96. Xiong W, Xiao MY, Zhang M, Chang F. Efficacy and safety of canagliflozin in patients with type 2 diabetes: A meta-analysis of randomized controlled trials. *Medicine*. 2016;95(48):e5473. doi: 10.1097/MD.0000000000005473.
97. Xu L, Li Y, Lang J, et al. Effects of sodium-glucose co-transporter 2 (SGLT2) inhibition on renal function and albuminuria in patients with type 2 diabetes: a systematic review and meta-analysis. *PeerJ*. 2017;5:e3405. doi: 10.7717/peerj.3405.
98. Yamani N, Usman MS, Akhtar T, Fatima K, Asmi N, Khan MS. Sodium-glucose co-transporter 2 inhibitors for the prevention of heart failure in type 2 diabetes: A systematic review and meta-analysis. *Eur J Prev Cardiol*. 2019:2047487319841936. doi: 10.1177/2047487319841936.
99. Yang X, Lai D, Zhong X, Shen H, Huang Y. Efficacy and safety of canagliflozin in subjects with type 2 diabetes: systematic review and meta-analysis. *Eur J Clin Pharmacol*. 2014;70:1149-1158. doi: 10.1007/s00228-014-1730-x.
100. Yang T, Lu M, Ma L, Zhou Y, Cui Y. Efficacy and tolerability of canagliflozin as add-on to metformin in the treatment of type 2 diabetes mellitus: a meta analysis. *Eur J Clin Pharmacol*. 2015;71:1325-1332. doi: 10.1007/s0028-015-1923-y.
101. Yang Y, Chen S, Pan H, et al. Safety and efficiency of SGLT2 inhibitor combining with insulin in subjects with diabetes: Systematic review and meta-analysis of randomized controlled trials. *Medicine*. 2017;96(21):e6944. Doi: 10.1097/MD.0000000000006944.
102. Zaccardi F, Webb DR, Htike ZZ, Youssef D, Khunti K, Davies MJ. Efficacy and safety of sodium-glucose co-transporter-2 inhibitors in type 2 diabetes mellitus: systematic review and network meta-analysis. *Diabetes Obes Metab*. 2016;18(8):783-794. doi: 10.1111/dom.12670.
103. Zelniker TA, Wiviott SD, Raz I, et al. SGLT2 inhibitors for primary and secondary prevention of cardiovascular and renal outcomes in type 2 diabetes: a systematic review and meta-analysis of cardiovascular outcome trials. *Lancet*. 2019;393(10166);31-39. doi: 10.1016/S0140-6736(18)32590-X.
104. Zhang M, Zhang L, Wu B, Song H, An Z, Li S. Dapagliflozin treatment for type 2 diabetes: a systematic review and meta-analysis of randomized controlled trials. *Diabetes Metab Res Rev*. 2014;30:204-221. doi: 10.1002/dmrr.2479.
105. Zhang XL, Zhu QQ, Chen YH, et al. Cardiovascular Safety, Long-Term Noncardiovascular Safety, and Efficacy of Sodium-Glucose Cotransporter 2 Inhibitors in Patients With Type 2 Diabetes Mellitus: A Systematic Review and Meta-Analysis With Trial Sequential Analysis. *J Am Heart Assoc.* 2018;7(2):e007165. doi: 10.1161/JAHA.117.007165.
106. Zhang L, Zhang M, Qingguo L, Tong N. Efficacy and safety of sodium-glucose cotransporter 2 inhibitors in patients with type 2 diabetes and moderate renal function impairment: A systematic review and meta-analysis. *Diabetes Res Clin Pract*. 2018;140:295-303. doi: 10.1016/j.diabres.2018.03.047.
107. Zhang YJ, Han SL, Sun XF, et al. Efficacy and safety of empagliflozin for type 2 diabetes mellitus: Meta-analysis of randomized controlled trials. *Medicine*. 2018;97(43):e12843. doi: 10.1097/MD.00000000000012843.
108. Zhang Q, Dou J, Lu J. Combinational therapy with metformin and sodium-glucose cotransporter inhibitors in management of type 2 diabetes: systematic review and meta-analyses. *Diabetes Res Clin Pract*. 2014;105(3):313-321. doi: 10.1016/j.diabres.2014.06.006.
109. Zhao D, Liu H, Dong P. Empagliflozin reduces blood pressure and uric acid in patients with type 2 diabetes mellitus: a systematic review and meta-analysis. *J Hum Hypertens*. 2019;33(4):327-339. doi: 10.1038/s41371-018-0134-2.
110. Zhao Y, Xu L, Tian D, et al. Effects of sodium-glucose co-transporter 2 (SGLT2) inhibitors on serum uric acid level: A meta-analysis of randomized controlled trials. *Diabetes Obes Metab*. 2018;20(2):458-462. doi: 10.1111/dom.13101.
111. Zheng SL, Roddick AJ, Aghar-Jaffar R, et al. Association Between Use of Sodium-Glucose Cotransporter 2 Inhibitors, Glucagon-like Peptide 1 Agonists, and Dipeptidyl Peptidase 4 Inhibitors With All-Cause Mortality in Patients with Type 2 Diabetes: A Systematic Review and Meta-analysis. *JAMA*. 2018;319(15):1580-1591. doi: 10.1001/jama.2018.3024.
112. Zhong M, Yang L, Chen X, Shen X. Therapeutic effect of canagliflozin on type 2 diabetes mellitus: a systematic review and meta-analysis. *Int J Clin Exp Med*. 2016;9(5):7807-7817.
113. Zhong X, Lai D, Ye Y, Yang X, Yu B, Huang Y. Efficacy and safety of empagliflozin as add-on to metformin for type 2 diabetes: a systematic review and meta-analysis. *Eur J Clin Pharmacol*. 2016;72:655-663. doi: 10.1007/s00228-016-2010-8.

Question

Quality

Rating

Study Q1 Q2 Q3 Q4 Q5 Q6 Q7 Q8 Q9 Q10 Q11 Q12 Q13 Q14 Q15 Q16

| Aronow (2017) | **Yes** | **Partial Yes** | **No** | **No** | **Yes** | **No** | **No** | **No** | **Yes** | **No** | **No** | **No** | **No** | **Yes** | **No** | **Yes** | **Critically low** |
| --- | --- | --- | --- | --- | --- | --- | --- | --- | --- | --- | --- | --- | --- | --- | --- | --- | --- |
| Dicembrini (2019) | **Yes** | **Yes** | **No** | **Partial Yes** | **Yes** | **Yes** | **No** | **No** | **Yes** | **No** | **No** | **No** | **No** | **Yes** | **Yes** | **Yes** | **Critically low** |
| Monami (2014) | **Yes** | **No** | **No** | **Partial Yes** | **Yes** | **Yes** | **No** | **No** | **Partial Yes** | **No** | **No** | **No** | **No** | **No** | **Yes** | **Yes** | **Critically low** |
| Radholm (2018) | **Yes** | **No** | **No** | **Partial Yes** | **Yes** | **Yes** | **No** | **Partial Yes** | **Yes** | **No** | **Yes** | **Yes** | **Yes** | **No** | **Yes** | **Yes** | **Moderate** |
| Storgaard (2016) | **Yes** | **Yes** | **No** | **Partial Yes** | **Yes** | **Yes** | **Yes** | **Yes** | **Yes** | **Yes** | **Yes** | **Yes** | **Yes** | **Yes** | **Yes** | **Yes** | **High** |
| Tang (2017) | **Yes** | **No** | **No** | **Partial Yes** | **No** | **No** | **No** | **Partial Yes** | **Yes** | **Yes** | **Yes** | **Yes** | **No** | **Yes** | **Yes** | **Yes** | **Low** |
| Tang (2018) | **Yes** | **No** | **No** | **Partial Yes** | **Yes** | **Yes** | **No** | **No** | **Yes** | **No** | **No** | **No** | **No** | **Yes** | **Yes** | **Yes** | **Critically low** |
| Wu (2016) | **Yes** | **No** | **No** | **Partial Yes** | **Yes** | **Yes** | **No** | **Partial Yes** | **Partial Yes** | **No** | **Yes** | **Yes** | **Yes** | **Yes** | **No** | **Yes** | **Moderate** |

Figure S1. A MeaSurement Tool to Assess systematic Reviews 2 (AMSTAR 2) quality assessment of included quantitative systematic reviews.

Readers interested in specific AMSTAR 2 checklist criteria may read the original *BMJ* publication found here: (<https://pubmed.ncbi.nlm.nih.gov/28935701/>).
