# Supplementary material for: The association of sodium‐glucose cotransporter 2 inhibitors with cancer: An overview of quantitative systematic reviews
Source: Endocrinol Diabetes Metab. 2020 May 20;3(3):e00145. doi: 10.1002/edm2.145 (PMC7375059; doi:10.1002/edm2.145)
Supplement: Supplementary file 2 — Appendix S2 [file EDM2-3-e00145-s002.docx]

**Appendix 2.**

**Page 2:** Figure S1. Skin cancers associated with sodium glucose co-transporter 2 use.

**Page 3:** Figure S2. Bladder cancer associated with sodium glucose co-transporter 2 use.

**Page 4:** Figure S3. Breast cancer associated with sodium glucose co-transporter 2 use.

**Page 5:** Figure S4. Renal cancer associated with sodium glucose co-transporter 2 use.

**Page 6:** Figure S5. Gastrointestinal cancers associated with sodium glucose co-transporter 2 use.

**Page 7:** Figure S6. Prostate cancer associated with sodium glucose co-transporter 2 use.

**Page 8:** Figure S7. Pulmonary cancer associated with sodium glucose co-transporter 2 use.

**Page 9:** Figure S8. Other specific cancers associated with sodium glucose co-transporter 2 use.


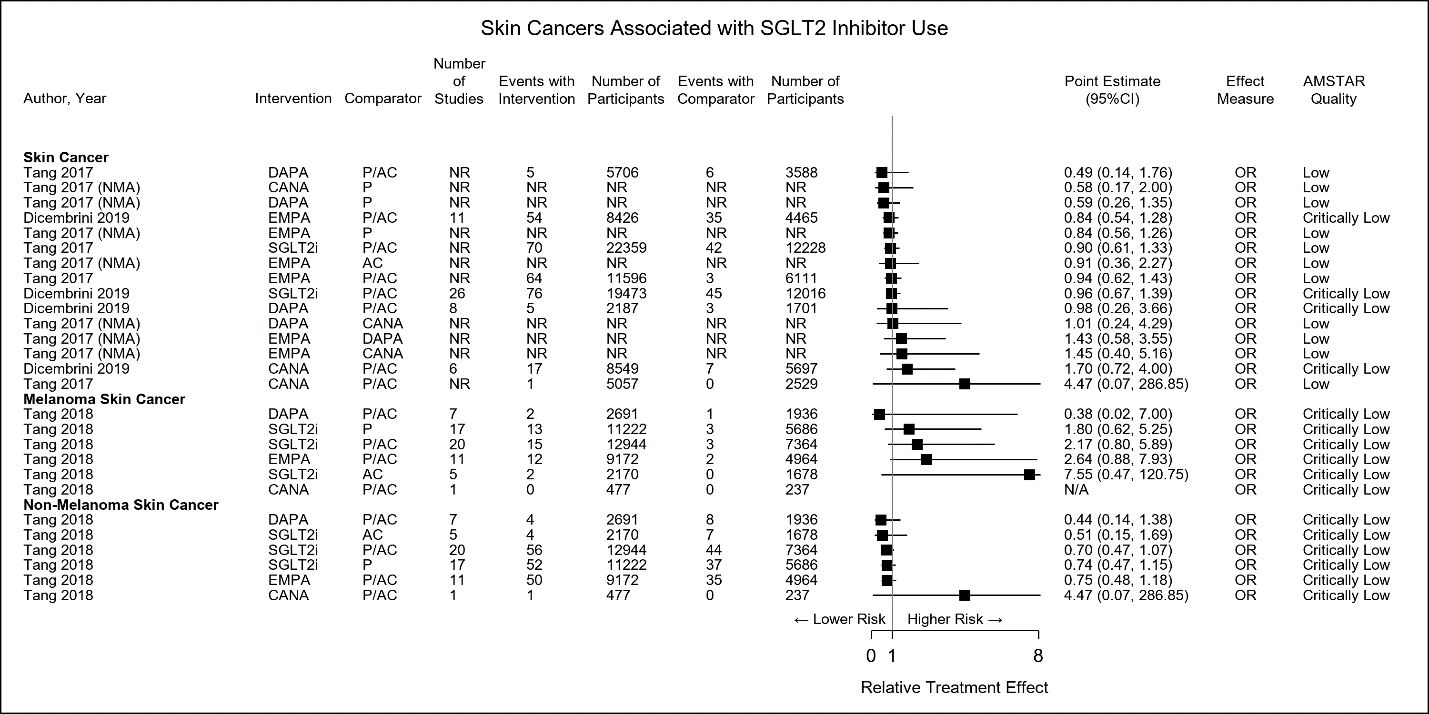


Figure S1. Skin cancers associated with sodium glucose co-transporter 2 use.

*SGLT2i=sodium glucose co-transporter 2 inhibitors (class effect), NMA=network meta-analysis NR=not reported*


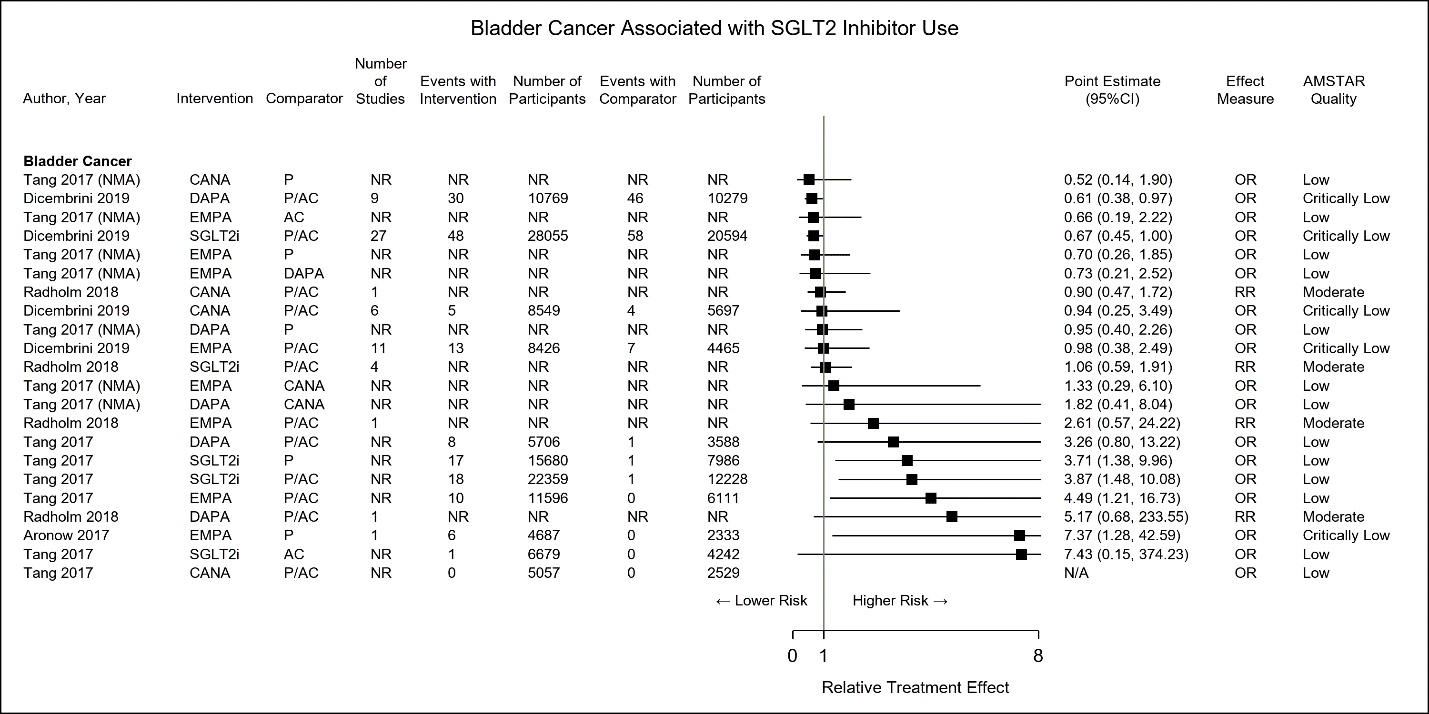


Figure S2. Bladder cancer associated with sodium glucose co-transporter 2 use.

*SGLT2i=sodium glucose co-transporter 2 inhibitors (class effect), NMA=network meta-analysis NR=not reported*


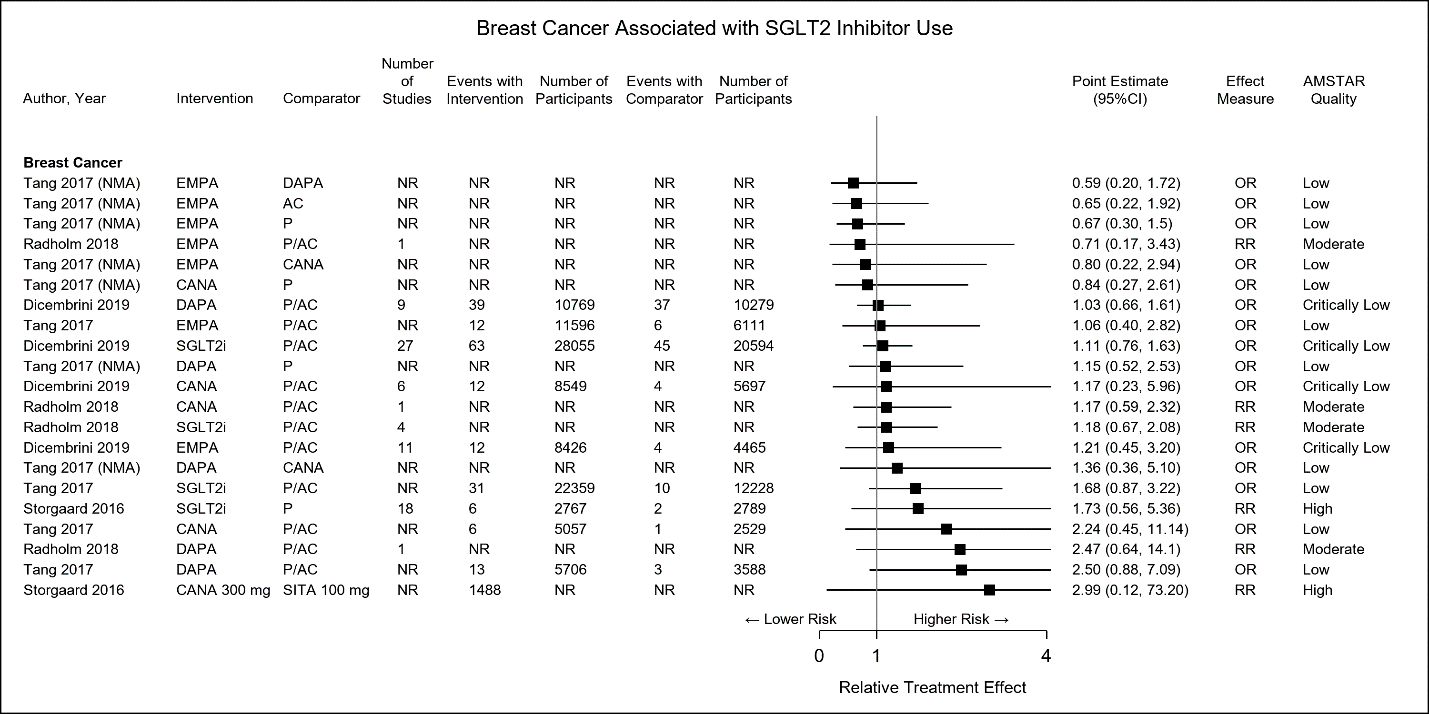


Figure S3. Breast cancer associated with sodium glucose co-transporter 2 use.

*SGLT2i=sodium glucose co-transporter 2 inhibitors (class effect), NMA=network meta-analysis NR=not reported*


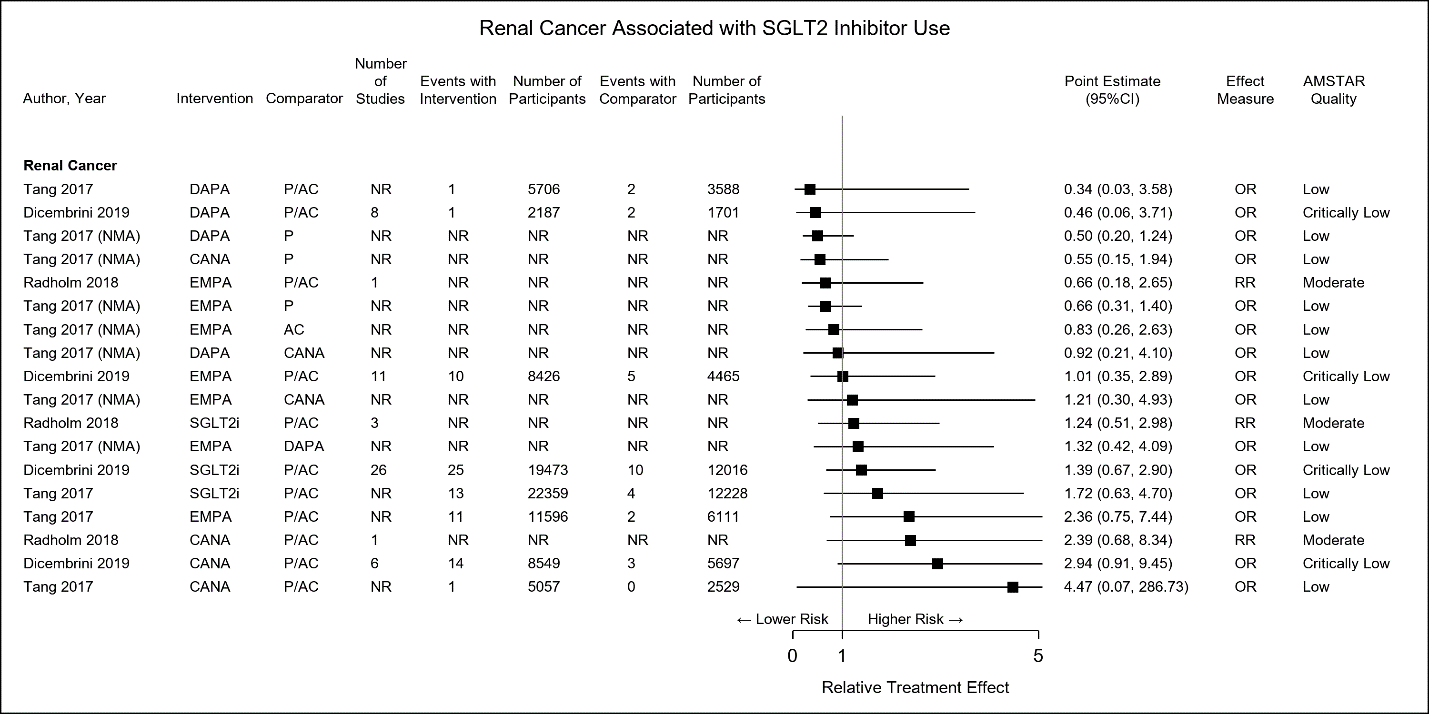


Figure S4. Renal cancer associated with sodium glucose co-transporter 2 use.

*SGLT2i=sodium glucose co-transporter 2 inhibitors (class effect), NMA=network meta-analysis NR=not reported*


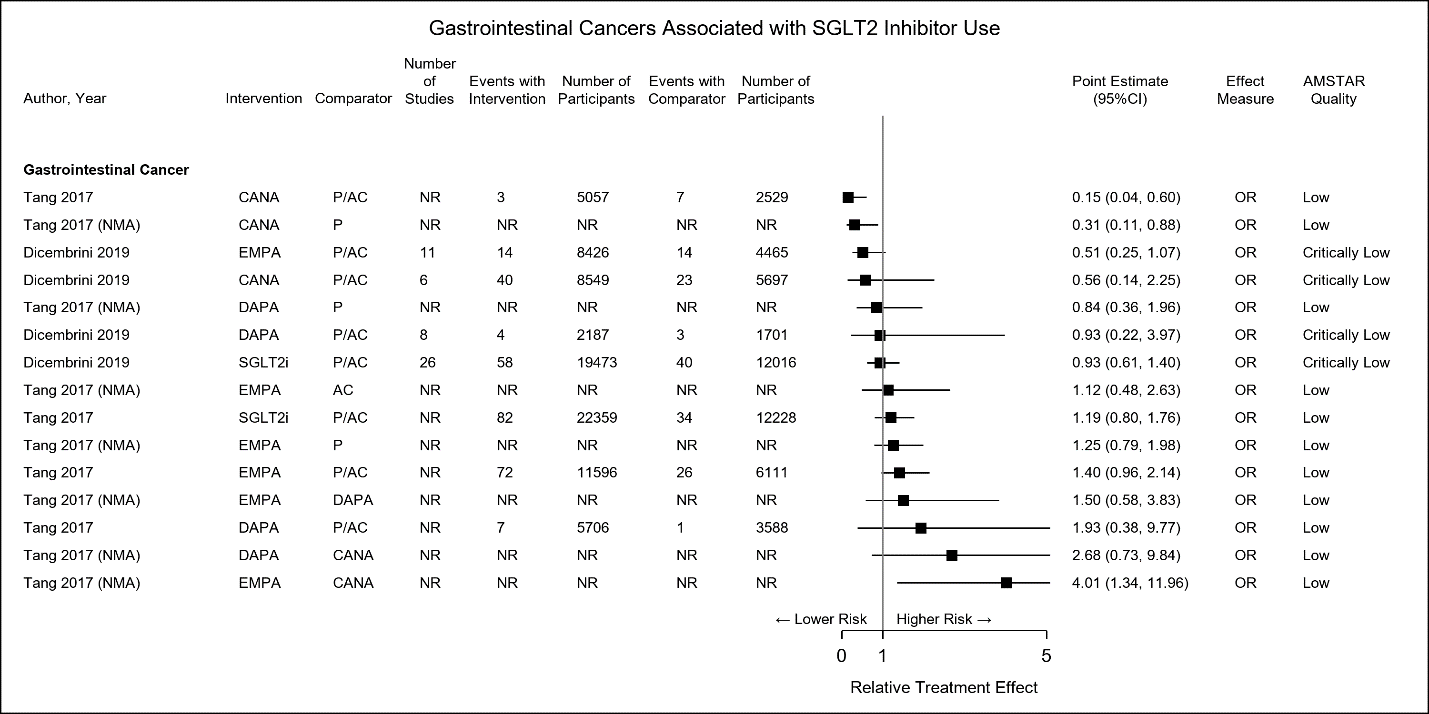


Figure S5. Gastrointestinal cancers associated with sodium glucose co-transporter 2 use.

*SGLT2i=sodium glucose co-transporter 2 inhibitors (class effect), NMA=network meta-analysis NR=not reported*


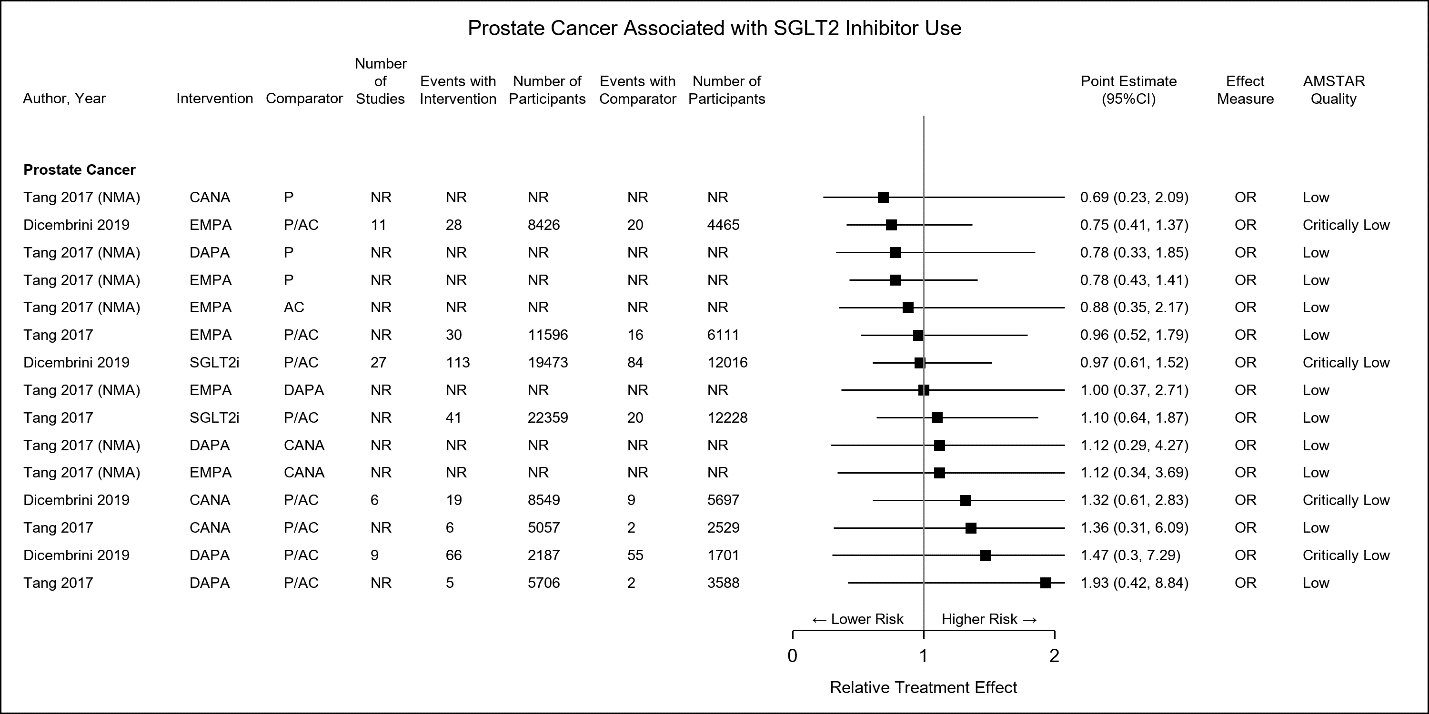


Figure S6. Prostate cancer associated with sodium glucose co-transporter 2 use.

*SGLT2i=sodium glucose co-transporter 2 inhibitors (class effect), NMA=network meta-analysis NR=not reported*


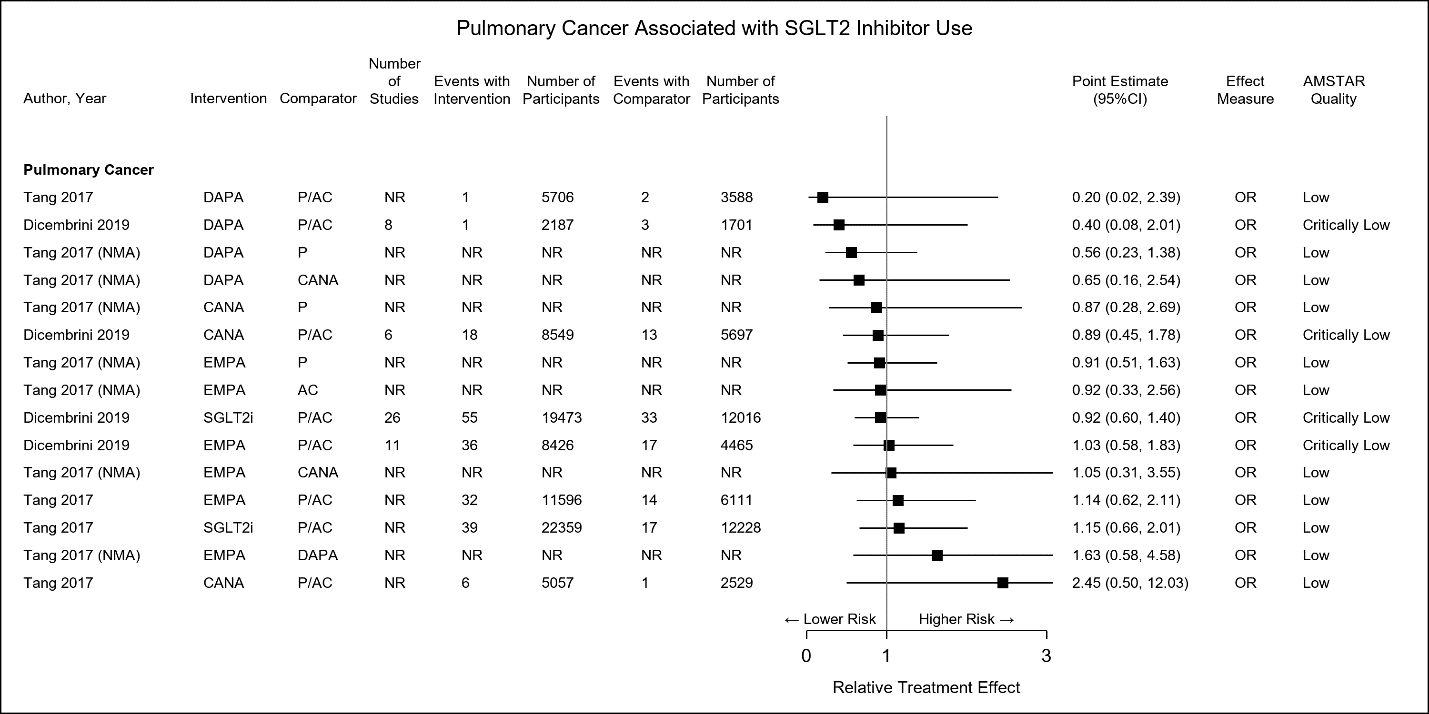


Figure S7. Pulmonary cancer associated with sodium glucose co-transporter 2 use.

*SGLT2i=sodium glucose co-transporter 2 inhibitors (class effect), NMA=network meta-analysis NR=not reported*


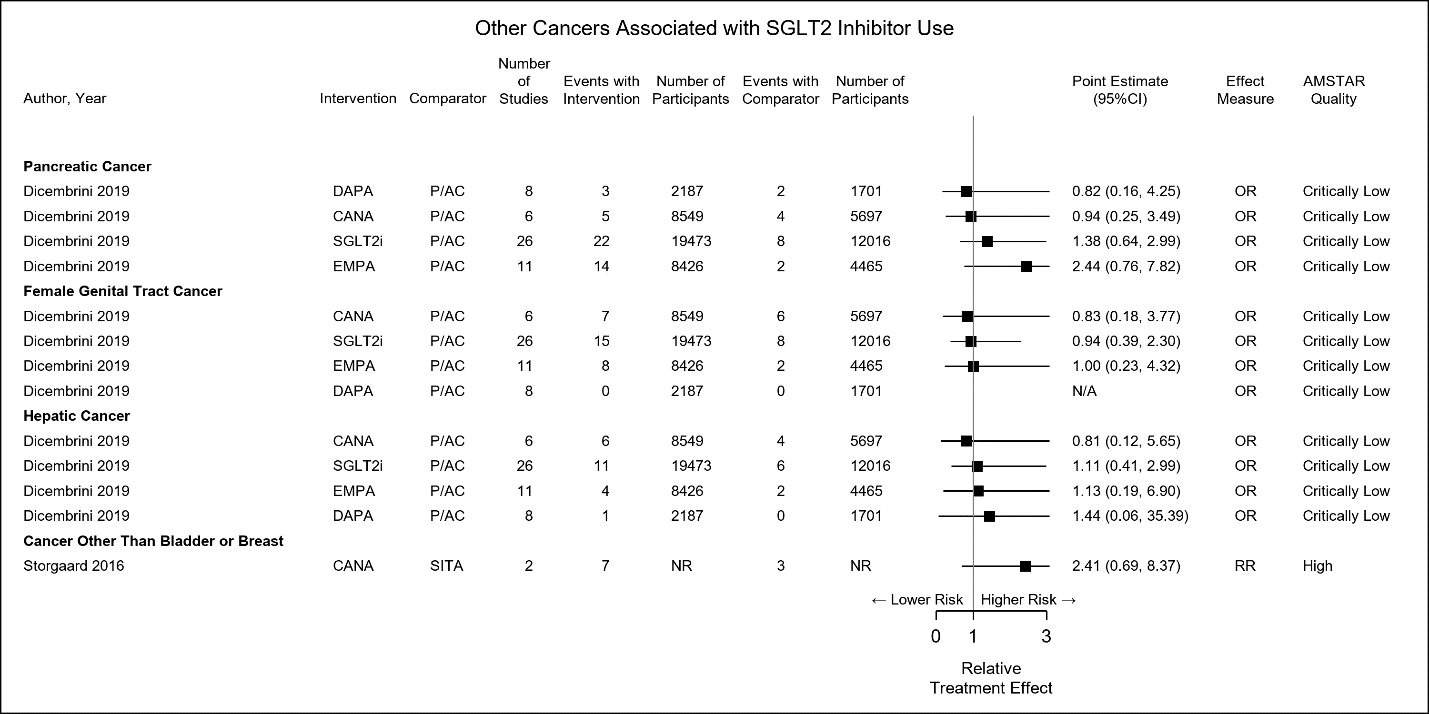


Figure S8. Other specific cancers associated with sodium glucose co-transporter 2 use.

*SGLT2i=sodium glucose co-transporter 2 inhibitors (class effect)NR=not reported*
